# Hereditary Angioedema Attacks in Patients Receiving Long-Term Prophylaxis: A Systematic Review

**DOI:** 10.1007/s12016-024-09006-1

**Published:** 2024-11-07

**Authors:** Hilary J. Longhurst, Mauro Cancian, Vesna Grivcheva-Panovska, Majed Koleilat, Markus Magerl, Sinisa Savic, Marcin Stobiecki, Raffi Tachdjian, Bridget Healy, Christopher M. Yea, Paul K. Audhya, Laurence Bouillet

**Affiliations:** 1 Department of Immunology, Auckland City Hospital, Te Toka Tumai and University of Auckland, Auckland, New Zealand; 2 Department of Systems Medicine, University Hospital of Padua, Padua, Italy; 3 University Clinic of Dermatology, School of Medicine, University Saints Cyril and Methodus, Skopje, North Macedonia; 4 Deaconess Clinic, Evansville, IN USA; 5 Angioedema Center of Reference and Excellence (ACARE) Institute of Allergology, Charité - Universitätsmedizin Berlin, Corporate Member of Freie Universität Berlin and Humboldt-Universität Zu Berlin, Berlin, Germany; 6 Fraunhofer Institute for Translational Medicine and Pharmacology ITMP, Immunology and Allergology, Berlin, Germany; 7 University of Leeds, Saint James’s University Hospital, Leeds, UK; 8 Department of Clinical and Environmental Allergology, Jagiellonian University Medical College, Krakow, Poland; 9 Department of Pediatrics, David Geffen School of Medicine, University of California, Los Angeles, CA USA; 10 ApotheCom, San Francisco, CA USA; 11 KalVista Pharmaceuticals, Salisbury, UK; 12 KalVista Pharmaceuticals, Cambridge, MA USA; 13 Grenoble Alpes University, CNRS, UMR 5525, VetAgro Sup, Grenoble INP, National Reference Center for Angioedema (CREAK), CHU Grenoble Alpes, TIMC, Grenoble, France

**Keywords:** Attack-free rate, Hereditary angioedema, Long-term prophylaxis, On-demand therapy, Systematic review

## Abstract

Long-term prophylaxis (LTP) has been shown to reduce the frequency of hereditary angioedema (HAE) attacks; however, attacks occurring in patients receiving LTP have not been well characterized. The objective of this systematic review was to evaluate the proportion of type I/II HAE (HAE-C1INH) patients who experience attacks while receiving LTP, the characteristics of these attacks, and associated on-demand therapy use. A systematic search was conducted in PubMed to identify studies reporting LTP use with plasma-derived C1 inhibitor (pdC1INH), lanadelumab, berotralstat, androgens, or antifibrinolytics in patients with HAE-C1INH. Forty-five primary studies met the inclusion criteria. In phase 3 trials, attack-free rates were 40% for subcutaneous pdC1INH 60 IU/kg twice weekly at 16 weeks, and 44% for lanadelumab 300 mg every second week at 6 months (77% during steady-state [days 70–182]); there was no difference in attack-free rate for berotralstat 150 mg versus placebo at 24 weeks. Phase 3 studies reported a lower average attack severity with subcutaneous and intravenous pdC1INH versus placebo. With lanadelumab and berotralstat, the prophylactic treatment effect was more pronounced in peripheral attacks than in abdominal and laryngeal attacks. Laryngeal attacks accounted for 2%-7% of all attacks in observational and interventional studies, regardless of the LTP agent received. On-demand therapy was used in 49%-94% of attacks occurring in the presence of LTP. In conclusion, patients receiving LTP experienced attacks in all anatomic locations, including the larynx. Most attacks were treated with on-demand therapy, although outcomes were not reported. Access to on-demand therapy remains essential for all people with HAE-C1INH.

**Supplementary Information:**

The online version contains supplementary material available at 10.1007/s12016-024-09006-1.

## Introduction

Hereditary angioedema (HAE) is a rare genetic disease resulting in deficiency (type I) or dysfunction (type II) of the C1 inhibitor protein (HAE-C1INH) and subsequent uncontrolled activation of the kallikrein kinin system, leading to unpredictable and often debilitating attacks of tissue swelling [1–4]. Since 2008, several long-term prophylactic (LTP) agents have been approved by the United States Food and Drug Administration and European Medicines Agency, including intravenous (IV) and subcutaneous (SC) plasma-derived C1INH (pdC1INH) replacement, the monoclonal plasma kallikrein-targeting antibody lanadelumab, and the oral small-molecule plasma kallikrein inhibitor berotralstat [2, 5]. Late phase investigational agents include garadacimab, a monoclonal antibody against factor XII, and donidalorsen, an antisense oligonucleotide that inhibits the production of plasma prekallikrein [3], but these agents have yet to receive approval in any country. HAE management guidelines indicate that attenuated androgens may also be used where non-androgen LTP options are not available [1, 2], while antifibrinolytics should only be considered for patients in whom the use of attenuated androgens is contraindicated [1]. The approval status of androgens and antifibrinolytics varies internationally [1, 2, 5, 6].

LTP agents have been demonstrated to reduce the frequency of HAE attacks [7–10]. In pivotal phase 3 trials, a ≥ 90% reduction in the frequency of attacks from baseline was reported in 67% of patients who received lanadelumab 300 mg every second week (Q2W) (*P* < 0.001 versus placebo) [8] and in 23% of patients who received berotralstat 150 mg once daily (QD) (not statistically significant versus placebo) [10]; 58% of patients who received SC pdC1INH 60 IU/kg twice weekly had a ≥ 90% reduction in frequency of attacks versus placebo (*P* value not reported) [9]. However, the characteristics of attacks that occur in the presence of LTP and the use of on-demand therapy to treat these attacks are not well understood.

The overarching goal of HAE treatment is to normalize patients' lives by achieving complete disease control [2, 4]. HAE treatment guidelines note that the availability of modern LTP agents, alongside personalized disease management, may facilitate the achievement of this goal (i.e., achieving an attack-free status) [1, 2, 4]. The purpose of this systematic review is to report the proportion of patients with HAE-C1INH receiving LTP who achieved the stated treatment goal of an attack-free status in phase 3 randomized controlled trials (RCTs), and the attack-free rates reported in open-label extension and real-world evidence studies across a longer treatment duration. This review also reports the attack characteristics (i.e., attack severity, duration, and location) and use of on-demand therapy in patients who continued to experience attacks while receiving LTP.

## Methods

Search procedures were established a priori in an operational protocol and followed the Preferred Reporting Items for Systematic Reviews and Meta-Analyses (PRISMA) guidelines. The operational procedure and full search strategy are available in the online supplement.

### Search Strategy and Inclusion/Exclusion Criteria

A systematic search was conducted in PubMed to identify peer-reviewed publications in English since January 1, 2002, that reported LTP use in patients with HAE-C1INH with one of the following drugs: IV C1INH, SC C1INH, lanadelumab, berotralstat, androgens (including danazol, stanozolol, oxandrolone, or methyltestosterone), antifibrinolytics (including aminocaproic acid or tranexamic acid [TA]), or any investigational LTP agent with published phase 2 or 3 clinical data (including garadacimab and donidalorsen). The initial search was conducted on May 17, 2022, and was updated on May 15, 2023. Reference lists of included articles, as well as reviews, editorial pieces, and other relevant publications, were searched for additional articles meeting inclusion criteria.

RCTs, nonrandomized interventional and open-label studies, registry studies, prospective and retrospective cohort studies, chart reviews, and case series were eligible for inclusion. Depending on the study design, comparators could include either patients receiving placebo, patients receiving on-demand therapy only, or patient baseline measurements (i.e., before initiation of LTP). Studies that did not clearly report on HAE-C1INH or that reported patients taking only on-demand therapy or only short-term prophylaxis were excluded. Individual case reports and articles where the full text was not available were also excluded.

The primary outcome of interest was the proportion of patients receiving LTP who achieved attack-free status across the specified treatment duration. Secondary outcomes included the proportion of attacks by attack location (e.g., peripheral, abdominal, facial, and laryngeal), attack severity, attack duration, and the proportion of attacks treated with on-demand therapy. A full list of the outcomes used for inclusion is available in the online supplement.

### Study Selection, Data Extraction, and Quality Assessment

All identified articles were manually reviewed at the title/abstract level. If deemed suitable, the full text was initially assessed for inclusion by one reviewer. Eligibility was then checked by a second reviewer. Disagreements regarding the inclusion of full-text articles were resolved through discussion with an additional reviewer, until consensus was reached. For each included article, data were extracted on the publication characteristics and study design, the treatment characteristics (LTP agent and dose, duration of treatment, and comparator), the proportion of attack-free patients in each treatment group, the attack characteristics (attack frequency, location, duration, and severity at baseline and after commencing study treatment), and on-demand therapy use. Assessments for risk of bias were performed for included studies using the Risk of Bias 2 tool [11] for RCTs (rated as high, moderate, or low risk of bias) and the Newcastle–Ottawa Scale for observational studies (rated on a star system on the selection of study groups, the comparability of the groups, and the ascertainment of the outcome of interest) [12]. All transcribed data and risk of bias assessments performed by one reviewer were checked for accuracy and completeness by a second reviewer, with any disagreements being resolved through discussion until consensus was reached.

### Data Reporting and Feasibility Assessment for Meta-analyses

Data on outcomes of interest were collectively summarized, reporting the findings of high-quality, phase 3, placebo-controlled RCTs first, followed by phase 2 trials and large extension or registry studies in which data were prospectively collected. Percentages extracted from publications were rounded to the nearest whole number. Means, standard deviations (SDs), standard errors (SEs), and confidence intervals (CIs) were rounded to the nearest first decimal. *P* values were included as reported in the source publication. A feasibility assessment for conducting meta-analyses was conducted. However, because of substantial limitations of data availability in RCTs and real-world evidence studies on the endpoints of interest, a meta-analysis was not considered feasible.

## Results

**Fig. 1:**
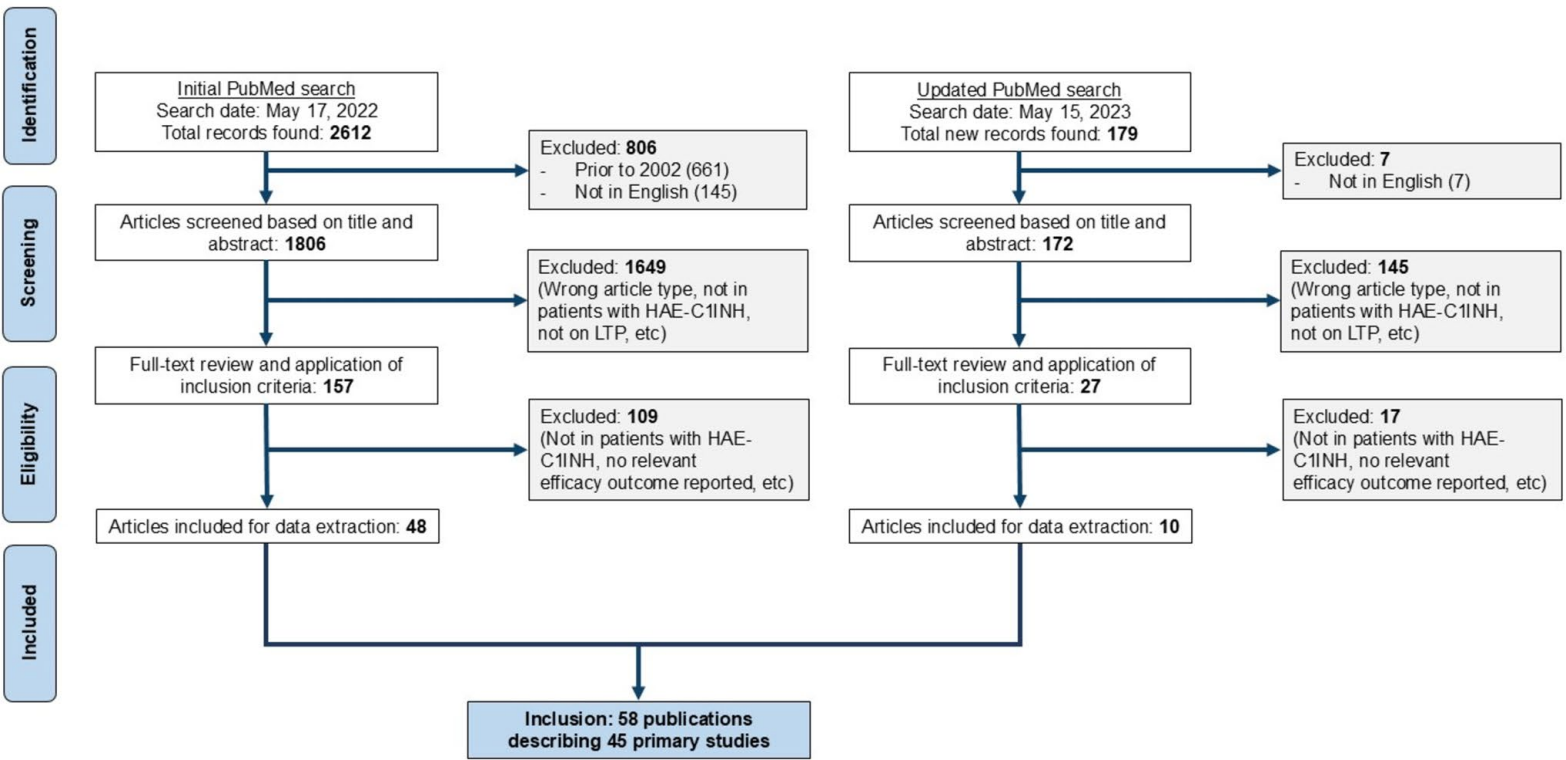
PRISMA flow diagram of included studies. HAE-C1INH, hereditary angioedema with deficiency (type I) or dysfunction (type II) of the C1 inhibitor protein; LTP, long-term prophylaxis

The initial PubMed search returned 2612 records, and the updated search identified an additional 179 records (Fig. 1)*.* Of these, 58 publications describing 45 primary studies published between January 1, 2002, and May 15, 2023, met the inclusion criteria and were included in the present review*.* Of the 45 primary studies, 13 were RCTs (8 were phase 3 RCTs, 4 were phase 2 RCTs, and 1 was a phase 1b RCT), 7 were open-label studies, and 25 were observational studies (12 retrospective case series or chart reviews, 10 prospective cohort or registry studies, and 3 patient surveys). In the quality assessment of the 45 primary studies, 10 RCTs were assessed as having a low bias risk and 2 had a moderate bias risk; 1 non–placebo controlled pediatric RCT was assessed as having a high bias risk (Table S1. Supplementary Material). Of the 25 observational studies, 12 were considered high quality (≥ 6 of 8 stars), 7 moderate quality (4 or 5 stars), and 6 low quality (≤ 3 stars).

### Proportion of Patients Who Received LTP Who Were Attack-free

For pdC1INH, 38% and 40% of participants aged ≥ 12 years were attack free after 16 weeks treatment with SC pdC1INH 40 IU/kg (*n* = 43) and 60 IU/kg (*n* = 43) twice weekly, respectively, in the phase 3 COMPACT trial (*N* = 90) [9] (Table 1), and 44% of participants were attack free after ≥ 52 weeks with SC pdC1INH 60 IU/kg twice weekly in the COMPACT open-label extension (*N* = 63) [13] (Table 2). No placebo-controlled RCT data reporting the attack-free rate were identified for IV pdC1INH. However, in the phase 3 CHANGE-3 open-label extension (*N* = 146), 35% of participants were attack free with IV pdC1INH 1000 U every 3 to 7 days across a median treatment period of 248 days [14] (Table 2). The efficacy of IV pdC1INH was directly correlated with the interval between administration (twice weekly versus once weekly) but not with historical attack frequency or duration of treatment [14]. Simulated pharmacokinetic modeling of functional C1INH from the COMPACT phase 2 trial predicted lower peak-to-trough ratios and higher trough values following SC pdC1INH administration than with IV pdC1INH [15], and may explain why a trend toward greater freedom of attacks was reported with long-term use of SC pdC1INH 60 IU/kg twice weekly (44%, *N* = 63) versus IV pdC1INH 1000 U every 3 to 7 days (35%, *N* = 146) in open-label extension studies [9, 14].

**Table 1:** Proportion of patients who received long-term prophylaxis who were attack free in phase 3 randomized placebo-controlled trials

Study identifier	Duration of treatment	Study population	No. of patients	LTP agent and comparator	Proportion of attack-free patients, %
NCT01912456 (COMPACT) [9]	2 × 16-weeks	Adolescents aged ≥ 12 years and adults	90	SC pdC1INH 40 IU/kg twice weekly	38
				SC pdC1INH 60 IU/kg twice weekly	40
				Placebo (Group 1)	9
				Placebo (Group 2)	0
NCT02586805 (HELP) [8, 16]	26 weeks	Adolescents aged ≥ 12 years and adults	125	Lanadelumab 150 mg Q4W	39
				Lanadelumab 300 mg Q4W	31
				Lanadelumab 300 mg Q2W	44
				Placebo	2
		Adolescents aged ≥ 12 years and adults (post hoc analysis of steady state [days 70–182])	120	Lanadelumab 150 mg Q4W	54
				Lanadelumab 300 mg Q4W	45
				Lanadelumab 300 mg Q2W	77
				Placebo	3
NCT04656418 (VANGUARD) [17]	6 months	Adolescents aged ≥ 12 years and adults	64	Garadacimab 200 mg QM^a^	62
				Placebo	0

*LTP*, long-term prophylaxis; *pdC1INH,* plasma-derived C1 inhibitor; *Q2W,* every 2 weeks; *Q4W,* every 4 weeks; *QM*, once monthly; *SC,* subcutaneous. ^a^Patients received a 400-mg SC loading dose on day 1

**Table 2:** Proportion of patients who received long-term prophylaxis who were attack free in phase 3 non-placebo-controlled trials and real-world observational studies

Study identifier/design	Duration of treatment	Study population	No. of patients	LTP agent and comparator	Proportion of attack-free patients, %
NCT02316353 (COMPACT OLE) [13, 18, 19]	52–140 weeks	Children aged ≥ 6 years and adults^b^	63	SC pdC1INH 60 IU/kg twice weekly	44
		Subgroup: adults aged ≥ 65 years	10	SC pdC1INH 40 or 60 IU/kg twice weekly	30
		Subgroup: children aged ≥ 6 to < 18 years	10	SC pdC1INH 40 or 60 IU/kg twice weekly	10
Japanese open-label study [20]	16 weeks	Adolescents aged ≥ 12 years and adults	9	SC pdC1INH 60 IU/kg twice weekly	67
NCT00462709 (CHANGE-3 OLE) [14, 21, 22]	248 days (median)	Children aged ≥ 1 year and adults	146	IV pdC1INH 1000 U every 3–7 days	35
		Subgroup: children aged ≥ 2 to < 18 years	23	IV pdC1INH 1000 U every 3–7 days	22
		Subgroup: pregnant women	11	IV pdC1INH 1000 U every 3–7 days	55
NCT02052141 (Pediatric crossover trial) [23]	2 × 12-weeks	Children aged ≥ 6 to < 12 years	12	IV pdC1INH 500 U every 3–4 days	25
				IV pdC1INH 1000 U every 3–4 days^a^	33
NCT02741596 (HELP OLE) [24]	33 months (median)	Adolescents aged ≥ 12 years and adults	209	Lanadelumab 300 mg Q2W^c^	37
Canadian retrospective chart review [25]	12 months	Patients aged 24–74 years commencing lanadelumab	12	Lanadelumab 300 mg Q2W^d^	25
Single-center retrospective chart review [26]	36 weeks (median)	Patients aged 21–55 years commencing lanadelumab	9	Lanadelumab 300 mg Q2W or Q4W	56
US-HAEA patient survey [27]	NR	Patients from the US-HAEA registry	344^e^	Attenuated androgens^f^	26^e^
Chinese retrospective cohort [28]	1 year	Patients aged ≥ 11 years of age from China	74	Danazol^f^	34^g^
German retrospective chart review [29]	11 years (mean)	Patients aged 15–74 years from Germany/Denmark	118	Danazol^f^	24
Swiss retrospective cohort [30]	1 year	Patients aged ≥ 5 years from Switzerland	26	Danazol^f^	38
			10	Tranexamic acid^f^	20
CREAK retrospective chart review [31]	6 months	Patients aged ≥ 16 years from France	12	Tranexamic acid^f^	8

*CREAK,* National Reference Centre for Angioedema (France); *IV,* intravenous; *LTP*, long-term prophylaxis; *NR,* not reported; *OLE,* open-label extension; *pdC1INH,* plasma-derived C1 inhibitor; *Q2W,* every 2 weeks; *Q4W,* every 4 weeks; *SC*, subcutaneous; *US-HAEA,* United States Hereditary Angioedema Association

^a^IV pdC1INH 1000 U every 3 or 4 days exceeds the recommended dose for children < 12 years of age [32]. ^b^Post hoc analysis in patients randomly assigned to the SC pdC1INH 60 IU/kg treatment arm. ^c^In rollover patients, a single dose of lanadelumab 300 mg was received at study entry and until the patient experienced their first attack, following which the patient received lanadelumab 300 mg Q2W. Non-rollover patients received lanadelumab 300 mg Q2W from study entry onward. ^d^One patient switched from lanadelumab 300 mg Q2W to Q4W. ^e^Percentage of attack-free patients among 344 patients who received attenuated androgens at the time of the survey. ^f^The dosage and dosing frequency of each LTP were variable or were not reported. ^g^The study results showed the outcome as the proportion of patients who had ≤ 1 attack per year rather than the proportion of patients who were attack free

For lanadelumab, 39%, 31%, and 44% of participants aged ≥ 12 years were attack free after 6 months of treatment (150 mg every 4 weeks [Q4W; *n* = 28], 300 mg Q4W [*n* = 29], and 300 mg Q2W [*n* = 27], respectively) in the phase 3 HELP trial (*N* = 125) [8] (Table 1). In a post hoc analysis of steady state between day 70 and day 182 (*n* = 120), the attack-free rate reached 77% with use of lanadelumab 300 mg Q2W (*n* = 26) [8, 16]. In the HELP open-label extension (*N* = 209), 37% of patients were attack free with lanadelumab over a median 33 months of treatment [24] (Table 2). Similar attack-free rates were reported for rollover patients (defined as patients who participated in the HELP RCT and who continued into the HELP open-label extension) and non-rollover patients (defined as newly enrolled patients who had not participated in the HELP RCT) [24]. The attack-free rate was 35% in rollover patients (*n* = 106), who received a single dose of lanadelumab 300 mg at entry into the extension study and only received regular lanadelumab dosing (300 mg Q2W) after experiencing their first HAE attack in the study. Non-rollover patients (*n* = 103) received lanadelumab 300 mg Q2W from study entry onwards and had an attack-free rate of 37% [24]. Data on the real-world attack-free rate for lanadelumab are limited: in a single-center retrospective chart review, 56% of 9 patients were attack free with lanadelumab 300 mg Q2W or Q4W over a median 36 weeks of treatment [26]. However, only 25% of 12 patients were attack free following 12 months of treatment with lanadelumab 300 mg Q2W in a retrospective chart review of patients from Canada [25].

For berotralstat, 0%, 43%, 21%, and 39% of participants aged ≥ 18 years were attack free following 28 days of treatment (62.5 mg [*n* = 7], 125 mg [*n* = 14], and 250 mg [*n* = 15], and 350 mg [*n* = 18] QD, respectively) in the phase 2 APeX-1 trial (*N* = 75) [33]. The absence of a significant difference in attack-free rates was reported between participants who received berotralstat 150 mg QD (*n* = 40), berotralstat 110 mg QD (*n* = 41), and placebo (*n* = 39) across 24 weeks in the phase 3 APeX-2 trial (*N* = 121) [10]. However, the absolute attack-free rates in the APeX-2 phase 3 RCT and the subsequent APeX-S open-label extension were not reported [10, 34, 35].

No RCTs that reported attack-free rates were identified in the search window for patients who received LTP with androgens or TA. In observational studies, 24%-38% of patients who received danazol and ≤ 20% of patients who received TA for ≥ 1 year were attack free [28–31] (Table 2).

### Attack Location in Patients Who Received LTP

Attacks at all anatomic locations, including laryngeal attacks, continued to occur in patients who received LTP, regardless of the LTP agent [8–10, 18, 24, 30, 31, 36–40]. Of note, both interventional and observational studies reported that laryngeal attacks accounted for between 2%-7% of all attacks in patients who received LTP with pdC1INH, lanadelumab, danazol, or TA [8, 9, 18, 24, 36]. The occurrence of laryngeal attacks in patients receiving LTP with berotralstat was also reported in phase 2 and phase 3 trials [10, 34, 35]; however, the proportion of attacks with laryngeal involvement for each treatment group was not reported.

In phase 3 studies of lanadelumab and berotralstat, there appeared to be a differential reduction in peripheral attacks compared with abdominal and laryngeal attacks [8, 10, 24]. In the HELP RCT, the proportion of peripheral attacks decreased from 72% (56 of 78 attacks) during the 4-to-8 week run-in period to 43% (20 of 46 attacks) during the 26-week treatment period for participants who received lanadelumab 300 mg Q2W. Conversely, the proportion of abdominal attacks increased from 27% (21 of 78 attacks) during the run-in period to 50% (23 of 46 attacks) during the treatment period, and the proportion of laryngeal attacks increased (1% [1 of 78 attacks] during the run-in period to 7% [3 of 46 attacks] during the treatment period) [8], suggesting that the prophylactic effect is more pronounced in peripheral attacks than in abdominal and laryngeal attacks. Further, abdominal attacks were the most prevalent on-treatment attack location in participants who received lanadelumab (all dose levels and administration frequencies) in both the phase 3 HELP trial and open-label extension study (60% and 61%, respectively) over peripheral attacks (38% and 36%) and laryngeal attacks (3% and 4%) [8, 24]. In the phase 3 ApeX-2 trial there was a larger difference in the normalized monthly peripheral attack rate for berotralstat 150 mg QD versus placebo (0.5 vs 1.2) over the abdominal attack rate (0.2 vs 0.4), laryngeal attack rate (0.1 vs 0.2), and mixed-location attack free (0.6 vs 0.7) [10]. Baseline and on-treatment attack location data in patients receiving IV or SC pdC1INH were not reported in the pivotal phase 3 RCTs or open-label extension studies identified in this review [9, 13, 14, 41–44].

### Attack Severity and Duration in Patients Who Received LTP

A lower attack severity compared with placebo was reported in phase 3 placebo-controlled trials of SC and IV pdC1INH [9, 42]. In these trials, attack severity was assessed using an attack severity score based on a 3-point scale, with 1 indicating mild, 2 indicating moderate, and 3 indicating severe [9, 42]. The mean (SD) attack severity score was 1.6 (0.6) for SC pdC1INH 60 IU/kg twice weekly versus 1.9 (0.5) for placebo in the COMPACT trial (*P* value not reported) [9] and 1.3 (0.9) for IV pdC1INH 1000 U every 3 to 4 days versus 1.9 (0.4) for placebo in the LEVP2005-1 trial (*P* < 0.001) [42] (Table 3).

**Table 3:** Attack severity in patients who received long-term prophylaxis in phase 3 randomized placebo-controlled trials

First author, year of publication	Duration of treatment	No. of patients	Assessment of attack severity	LTP agent and dose	Attack severity, mean (SD) or n (%)		
					LTP	Placebo	*P* value
Longhurst, 2017 [9]; Li, 2019 [41]	2 × 16-weeks	90	Attack severity score, mean (SD)^a^	SC pdC1INH 40 IU/kg twice weekly	1.8 (0.6)	2.0 (0.5)	NR
				SC pdC1INH 60 IU/kg twice weekly	1.6 (0.6)	1.9 (0.5)	NR
Zuraw, 2010 [42]	2 × 12-weeks	24	Attack severity score, mean (SD)^a^	IV pdC1INH 1000 U every 3–4 days	1.3 (0.9)	1.9 (0.4)	< 0.001
Banerji, 2018 [8]	26 weeks	125	Patients with a maximum attack severity of ‘severe’, n (%)^b^	Lanadelumab 150 mg Q4W	5 (18)	14 (34)	0.18
				Lanadelumab 300 mg Q4W	4 (14)	14 (34)	0.02
				Lanadelumab 300 mg Q2W	2 (7)	14 (34)	0.02
Craig, 2023 [17]	6 months	64	Patients with a maximum attack severity of ‘severe’, n (%)^c^	Garadacimab 200 mg QM^d^	5 (13)	10 (42)	NR

*IV,* intravenous; *LTP*, long-term prophylaxis; *NR,* not reported; *pdC1INH,* plasma-derived C1 inhibitor; *Q2W*, every 2 weeks; *Q4W*, every 4 weeks; *QM*, once monthly; *SC*, subcutaneous; *SD*, standard deviation.

^a^Attack severity score was based on a 3-point scale, with 1 indicating mild, 2 indicating moderate, and 3 indicating severe. ^b^The difference from placebo was analyzed using Fisher exact test. ^c^Proportions were calculated with the number of patients in the treatment period for ≥ 30 days as the denominator (*n* = 39 for garadacimab; *n* = 24 for placebo). ^d^Participants received a 400-mg SC loading dose of garadacimab or placebo on day 1

The phase 3 trial for lanadelumab (HELP) reported a significantly lower number of moderate or severe attacks per month versus placebo (*P* < 0.0001 for lanadelumab 300 mg Q2W) and a lower proportion of patients with a maximum attack severity of ‘severe’ versus placebo [8] (Table 3). However, the change in average attack severity from baseline during treatment with LTP was not reported. Attack severity data were not published in the phase 3 APeX-2 trial for berotralstat [10, 35]. Although significant differences for attack severity versus placebo were reported for IV pdC1INH and lanadelumab (Table 3), none of the pivotal phase 3 RCTs identified in this review reported a significant reduction from baseline in attack severity for any LTP agent [8–10, 35, 42].

Findings from phase 3 non-placebo-controlled trials (e.g., open-label extension studies) and observational real-world studies generally supported a reduction in attack severity from baseline with LTP use [20, 23, 24, 27, 36, 37, 45–48], although many are limited by small sample sizes (Table S2). The mean (SD) number of severe attacks per month decreased from 7.2 (7.1) at baseline to 0.4 (1.4) after 6 months and 0.3 (0.7) after 12 months of treatment (*P* < 0.0001) in a real-world audit of 62 patients commencing on lanadelumab 300 mg Q2W in the United Kingdom [45]. Berotralstat 150 mg QD for 4 to 6 months reduced the mean attack severity compared with the 3-month period preceding commencement of berotralstat (*P* < 0.0001) in a real-world patient survey of 54 patients in the United Kingdom [47]. However, no significant reduction in attack severity was reported for patients who received LTP with C1INH, androgens, or TA, compared with patients who received treatment with on-demand therapy only in 448 patients participating in a prospective icatibant registry study [36]. In a prospective cohort of 49 patients from Australia, a numerically lower proportion of attacks were rated as severe or significant in patients who received lanadelumab compared with patients who received on-demand therapy only, but the proportion of attacks rated as severe or significant was similar or higher in patients who received SC or IV C1INH or danazol compared with patients receiving on-demand therapy only [48].

Data on attack duration in patients who received LTP were only reported in a minority of the interventional or observational studies included in this review [8, 9, 36, 37, 41, 42, 49]. A statistically significant difference in the mean (SD) attack duration was reported for IV pdC1INH versus placebo in the LEVP2005-1 trial (2.1 [1.1] vs 3.4 [1.4] days, respectively; *P* = 0.002) [42] (Table S3). However, no significant difference in attack duration was reported for any dose of lanadelumab versus placebo in the HELP trial (26.6 [22.7] hours for lanadelumab 300 mg Q2W versus 33.5 [23.4] hours for placebo; *P* = 0.330) [8] or SC pdC1INH versus placebo in the phase 3 COMPACT trial (1.6 [1.0] days for SC pdC1INH 60 IU/kg versus 1.6 [0.7] days for placebo; *P* value not reported) [41]. Attack duration data were not published in the phase 3 APeX-2 trial for berotralstat [10, 35]. No phase 3 RCTs identified in this study reported a significant reduction in attack duration from baseline for any LTP agent.

In observational studies (Table S4), a trend toward a shorter attack duration from baseline was reported for patients receiving attenuated androgens (median 1.5 days) and for antifibrinolytics (median 1.6 days) versus patients receiving on-demand treatment only (median 1.7 days) in a prospective cohort of 103 patients from Italy; however, the differences were not statistically significant [49]. No difference in attack duration was reported for attacks that occurred in patients who received LTP as opposed to patients who received treatment with on-demand therapy only in the icatibant registry study (8.0 vs 9.0 h, respectively; *P* = 0.543) [36]. When the data were analyzed per LTP agent, a shorter attack duration was reported in patients who received LTP with IV pdC1INH (mean 4.0 h; *P* = 0.041) versus patients who received treatment with on-demand therapy only but not in patients who received androgens (mean 8.0 h; *P* = 0*.*984) [36]. Patients who received TA had a longer attack duration than patients who received treatment with on-demand therapy only (11.6 vs 9.0 h; *P* = 0.016) [36].

### On-demand Therapy Use for Attacks in Patients Who Received LTP

Studies that reported on-demand therapy use showed that most attacks in patients who received LTP were treated with on-demand therapy [8, 13, 18, 34, 36, 37, 41, 50]. In pivotal phase 3 studies, 49%-68% of attacks in participants who received SC pdC1INH [41], 65%-83% of attacks in participants who received lanadelumab [8], and 82% of attacks in participants who received berotralstat [34] were treated with ≥ 1 dose of an on-demand therapy (Table 4). The on-demand agent administered varied across studies and included icatibant, ecallantide, IV C1INH replacement (plasma-derived or recombinant), or fresh frozen plasma [8, 34, 41].

**Table 4:** Proportion of attacks treated with on-demand therapy in patients who received long-term prophylaxis in phase 3 randomized placebo-controlled trials, open-label extensions studies, and real-world observational studies

First author, date of publication	Study details	No. of patients	LTP agent and comparator	Attacks treated with on-demand therapy, n/N (%)	Treated attacks requiring 2 doses of on-demand therapy, n/N (%)	Treated attacks requiring ≥ 3 doses of on-demand therapy, n/N (%)
Li, 2019 [41]	Phase 3 COMPACT RCT	90	SC pdC1INH 40 IU/kg twice weekly	99/145 (68)	7/99 (7)	0/99 (0)
			SC pdC1INH 60 IU/kg twice weekly	35/71 (49)	0/35 (0)	0/35 (0)
			Placebo^a^	779/975 (80)	60/779 (8)	29/779 (4)
Craig, 2022 [13]; Levy, 2020 [18]	Phase 3 COMPACT OLE	63	SC pdC1INH 60 IU/kg twice weekly	229/371 (62)	25/229 (11)	12/229 (5)
		10^b^	SC pdC1INH 40/60 IU/kg twice weekly	16/38 (42)	NR	NR
Rasmussen, 2016 [37]	Prospective cohort	6	IV pdC1INH 1000 U twice weekly	63/67 (94)	NR	NR
Banerji, 2018 [8]	Phase 3 HELP RCT	125	Lanadelumab 150 mg Q4W	55/84 (65)	NR	NR
			Lanadelumab 300 mg Q4W	87/105 (83)	NR	NR
			Lanadelumab 300 mg Q2W	38/46 (83)	NR	NR
			Placebo	506/572 (88)	NR	NR
Farkas, 2021 [34]	Phase 3 APeX-S OLE	227	Berotralstat 150 mg QD^c^	82%^d^	NR	NR
Aberer, 2017 [36]	Prospective registry	448	LTP (IV pdC1INH, androgens, TA)^e^	≥ 90% of 973 attacks^f^	9% of 973 attacks^f^	1% of 973 attacks^f^
			On-demand therapy only	≥ 92% of 2255 attacks^f^	8% of 2255 attacks^f^	1% of 2255 attacks^f^

*IV,* intravenous; *LTP*, long-term prophylaxis; *NR*, not reported; *OLE,* open-label extension; *pdC1NH,* plasma-derived C1 inhibitor; *Q2W*; every 2 weeks; *Q4W*; every 4 weeks; *QD*, once daily; *QM*, once monthly; *RCT*, randomized controlled trial; *SC*, subcutaneous; *TA*, tranexamic acid.

^a^Placebo calculated by combining two placebo groups. ^b^Pediatric subgroup analysis of children aged ≥ 6 to < 18 years. ^c^The study was initially designed to evaluate berotralstat 150 mg QD, but the protocol was amended to include a berotralstat 110 mg QD in selected patients. ^d^The absolute number of attacks and treated attacks were not reported. ^e^The dosage and dosing frequency of LTP were not reported. ^f^The study reported the proportion of patients who treated attacks with 1, 2, and ≥ 3 doses of on-demand therapy. The absolute number of treated attacks was not reported

Results of observational studies suggested that the proportion of attacks treated with on-demand therapy may be higher in real-world settings [36, 37] than the proportion reported in clinical trials [8, 34, 41], with data from the prospective icatibant registry reporting ≥ 90% of 973 attacks that occurred in patients who received LTP with C1INH, androgens, or TA were treated with ≥ 1 dose of an on-demand therapy [36].

A small proportion of treated attacks were treated with ≥ 2 doses of an on-demand therapy [13, 36, 41] (Table 4). In the phase 3 COMPACT trial, 8% of 779 treated attacks in participants who received placebo were treated with 2 doses of an on-demand therapy, compared with 7% of 99 treated attacks in participants who received LTP with SC pdC1INH 40 IU/kg twice weekly and none of 71 treated attacks in participants who received LTP with SC pdC1INH 60 IU/kg twice weekly [41]. In contrast, 11% of 229 treated attacks were treated with 2 doses of an on-demand therapy and 5% were treated with ≥ 3 doses in participants who received LTP with SC pdC1INH 60 IU/kg twice weekly in the COMPACT open-label extension [13]. In the prospective icatibant registry, 10% of 973 attacks were treated with ≥ 2 doses of an on-demand therapy in patients who received LTP with IV pdC1INH, androgens, or TA, compared with 8% of 2255 attacks in patients not receiving LTP treatment [36]. These findings suggest that no substantial differences existed in the proportion of attacks treated with an on-demand therapy, or the proportion of attacks treated with multiple doses of on-demand therapy, between patients who received LTP and patients treated with on-demand therapy alone.

### Investigational Agents

The phase 3 VANGUARD trial evaluating garadacimab reported that 62% of participants aged ≥ 12 years were attack free following 6 months of treatment with garadacimab 200 mg once monthly (*N* = 64) [17] (Table 1). A significantly lower number of moderate or severe attacks per month was reported for garadacimab versus placebo (*P* < 0.0001) and a numerically lower proportion of patients with maximum attack severity as ‘severe’ versus placebo (*P* value not reported) [17] (Table 3). The proportion of attacks which were treated with on-demand therapy was not reported in the phase 3 VANGUARD study. In a phase 2 trial of donidalorsen, 12 of 13 participants (92%) aged ≥ 18 years were attack-free with 80 mg Q4W during weeks 5 to 17 of treatment [51].

## Discussion

Clinical data and a recent Cochrane review have shown that LTP with C1INH replacement, lanadelumab, berotralstat, and danazol reduce the frequency of HAE attacks [7]. However, few reviews have reported the efficacy of LTP agents in terms of achieving the overarching treatment goal of an attack-free status or included the findings from real-world studies in efficacy assessments. This review confirms that a substantial proportion of patients who receive LTP do not achieve the goal of an attack-free status and experience attacks which are unpredictable with regard to severity, anatomical location, and duration.

Attack-free rates were generally low (< 45%) in patients who received LTP with IV or SC pdC1INH [9, 13, 14], berotralstat [33], danazol [28–30], and TA [30, 31]. Although attack-free rates in phase 3 RCTs were higher during the 16-week steady state period with lanadelumab 300 mg Q2W (77% between day 70 and day 182) [8] and across a 6-month treatment duration for garadacimab 200 mg QM (62%) [17], achieving an attack-free status remains elusive for many patients living with HAE-C1INH. Further, interventional and observational studies have reported that laryngeal attacks accounted for 2%-7% of all attacks in patients with HAE who received LTP [8, 9, 18, 24, 36] and deaths from asphyxiation during laryngeal attacks have been reported in patients receiving LTP with androgens [52]. These findings therefore have important implications for clinical practice: physicians and patients living with HAE need to be aware that no LTP agent has been demonstrated to provide complete protection against laryngeal attacks.

A reduction in attack severity with LTP use from baseline (i.e., prior to commencing LTP) was reported in early observational studies [23, 27, 45, 47]. However, there was insufficient evidence from phase 3 trials to support a reduction in attack severity from baseline with pdC1INH, lanadelumab, or berotralstat. Although a lower number of moderate or severe attacks per month compared with placebo was reported in phase 3 trials of lanadelumab [8], and garadacimab [17], these findings are likely a reflection of the overall reduction in attack frequency and do not provide direct evidence for a change in average attack severity for attacks occurring in the presence of LTP. The interpretation of attack severity and attack duration outcomes in phase 3 trials of nonandrogen LTP agents is further limited by a lack of assessment for the potentially confounding role of on-demand therapy administration. Early versus late administration of an effective on-demand therapy is well established to significantly shorten attack duration and reduce the time to attack resolution, irrespective of attack severity or location [1, 2], and there are no data suggesting that attacks occurring in the presence of LTP are mechanistically different from attacks in the absence of LTP. It is therefore possible that early administration of on-demand therapy is a more important factor in reducing attack duration and severity than the presence or absence of LTP [49].

Collectively, the findings of this systematic review provide support to international treatment guidelines recommending that all people with HAE should have immediate access to ≥ 2 doses of on-demand therapy and should treat attacks early after recognition of onset to arrest the progression of swelling and shorten the time to attack resolution, including patients receiving LTP [1, 2]. Indeed, interventional and observational studies confirmed that most attacks occurring in patients who received LTP were treated with on-demand therapy [8, 13, 18, 34, 36, 37, 41, 50], with a small proportion of attacks treated with ≥ 2 doses [13, 36, 41]. Despite guideline recommendations stating that patients receiving LTP must also have access to effective on-demand treatment for acute attacks [1, 2], there is some evidence that this is not consistently enforced [53]. This analysis reinforces the importance of unrestricted access to potentially lifesaving on-demand treatment.

Limitations of this systematic review include the restriction to articles published from 2002 onward and articles published in English. Studies published before 2002 reporting the efficacy of attenuated androgens, TA, and pdC1INH were therefore not included. Other limitations include the lack of head-to-head trials to directly compare efficacy between LTP agents, inconsistency in endpoint reporting between studies, differences in study populations, and the small number of participants and limited timepoints for data collection on attack symptoms in some observational studies. Reviews of outcome measures used to assess the efficacy of on-demand treatment and LTP report that no single outcome measure was utilized uniformly across all trials and recommended that future trials should endeavor to select outcome measures that are the most meaningful to patients [54, 55]. In a patient survey, the ‘proportion of attack-free patients’ was identified as a priority outcome of interest for patients [54]; however, only 20 of the 45 studies (44%) included in this systematic review reported the attack-free rate. Consistent reporting of attack characteristics at baseline and during treatment may clarify whether LTP has differential effects on attack location. As multiple LTP agents and on-demand therapies are in use, data on the efficacy of specific on-demand therapies used to treat attacks in patients receiving LTP with a similar or different mechanism of action could illuminate how LTP agents affect the efficacy and safety of on-demand therapies.

## Conclusions

This systematic review confirmed that achieving an attack-free status in many patients with HAE-C1INH remains a challenging goal. Although the use of LTP reduces attack frequency, patients continue to experience attacks in all anatomic locations, including potentially life-threatening laryngeal attacks. Most attacks that occurred in patients who received LTP were treated with ≥ 1 dose of an on-demand therapy, and unrestricted access to effective on-demand therapy remains essential for all people with HAE-C1INH, including patients receiving LTP.

## Supplementary Information

Below is the link to the electronic supplementary material.

Supplementary file1 (PDF 424 KB)

## Data Availability

No datasets were generated or analyzed during the current study.
