# Supplementary material for: Hereditary Angioedema Attacks in Patients Receiving Long-Term Prophylaxis: A Systematic Review
Source: Clin Rev Allergy Immunol. 2024 Nov 7;67(1-3):83–95. doi: 10.1007/s12016-024-09006-1 (PMC11638394; doi:10.1007/s12016-024-09006-1)
Supplement: Supplementary file 1 — (PDF 424 KB) [file 12016_2024_9006_MOESM1_ESM.pdf]

Hereditary Angioedema Attacks in Patients Receiving Long-Term Prophylaxis: A Systematic Review

Authors: Hilary J. Longhurst, MD, PhD,[1] Mauro Cancian, MD,[2] Vesna Grivcheva-Panovska, MD, PhD,[3] Majed Koleilat, MD,[4] Markus Magerl, MD, PhD,[5,6] Sinisa Savic, MD, PhD,[7] Marcin Stobiecki, MD,[8] Raffi Tachdjian, MD, MPH,[9] Bridget Healy, MBChB, MPH,[10] Christopher M. Yea, PhD,[11] Paul K. Audhya, MD,[12] Laurence Bouillet, MD, PhD[13]

Affiliations: [1]Department of Immunology, Auckland City Hospital, Te Toka Tumai and University of Auckland, Auckland, New Zealand; [2]Department of Systems Medicine, University Hospital of Padua, Padua, Italy; [3]University Clinic of Dermatology, School of Medicine, University Saints Cyril and Methodus, Skopje, North Macedonia; [4]Deaconess Clinic, Evansville, IN, USA; [5]Angioedema Center of Reference and Excellence (ACARE) Institute of Allergology, Charité - Universitätsmedizin Berlin, Corporate Member of Freie Universität Berlin and Humboldt-Universität zu Berlin, Berlin, Germany; [6]Fraunhofer Institute for Translational Medicine and Pharmacology ITMP, Immunology and Allergology, Berlin, Germany; [7]University of Leeds, Saint James's University Hospital, Leeds, UK; [8]Department of Clinical and Environmental Allergology, Jagiellonian University Medical College, Krakow, Poland; [9]Department of Pediatrics, David Geffen School of Medicine, University of California, Los Angeles, CA, USA; [10]ApotheCom, San Francisco, CA, USA; [11]KalVista Pharmaceuticals, Salisbury, UK; [12]KalVista Pharmaceuticals, Cambridge, MA, USA; [13]Grenoble Alpes University, CNRS, UMR 5525, VetAgro Sup, Grenoble INP, National Reference Center for Angioedema (CREAK), CHU Grenoble Alpes, TIMC, Grenoble, France

Search Protocol for the Systematic Review of Attacks in Patients With Type I/II Hereditary Angioedema Receiving Long-term Prophylactic Therapy

Background

Hereditary angioedema (HAE) is a rare genetic disease [1,2]. People living with HAE experience painful, debilitating, and disfiguring attacks of tissue swelling, which may be life-threatening depending on the location affected [1-3]. The underlying cause is a deficiency (type I) or dysfunction (type II) of the C1 esterase inhibitor protein (C1INH), which in turn leads to uncontrolled activation of the kallikrein kinin system and release of bradykinin, triggering fluid extravasation and edema [4].

European Medicines Agency– and US Food and Drug Administration (FDA)–approved drugs for long-term prophylaxis (LTP) in people living with HAE are plasma-derived C1 inhibitor proteins (Cinryze® and Haegarda® [sc-Berinert®]), the plasma kallikrein inhibitors lanadelumab (Takhzyro®, a monoclonal antibody) and berotralstat (Orladeyo®) [1, 2]. HAE clinical guidelines indicate that attenuated androgens (eg, danazol, stanozolol) may also be used as a second-line treatment where first-line LTP options are not available [1,2]. Antifibrinolytic therapy with tranexamic acid (Cyklokapron® and Lysteda®) or epsilon aminocaproic acid (Amicar®) are also used as a second-line treatment for patients in whom the use of attenuated androgens is contraindicated, although they are not recommended by most international guidelines [1] and approval of these agents varies internationally. A study evaluating garadacimab, an investigational monoclonal antibody against factor XII, recently reported positive phase 3 trial results [5], although garadacimab had yet to receive approval in any country.

People with HAE receiving LTP may continue to experience HAE attacks [6]. These attacks have been named ‘breakthrough attacks’ [7], although there is currently no scientific rationale to suggest that attacks which occur when receiving LTP are mechanistically different from attacks that occur in the absence of LTP. HAE clinical guidelines therefore recommend that all people living with HAE have immediate access to ≥2 doses of an effective on-demand treatment at all times, including

patients receiving LTP [1, 2]. This systematic literature review will assess attacks in people with HAE receiving LTP and thereby provide insight into the continued need for on-demand therapies.

Objectives

Primary objective

- To determine the proportion of people with HAE-C1INH receiving LTP who continue to experience attacks

Secondary objectives

- To characterize the nature of attacks in patients with HAE-C1INH receiving LTP (eg, attack severity, duration, and location in the body)
- To describe the characteristics of patients with HAE-C1INH who continue to experience attacks while receiving LTP
- To report on the use of on-demand therapies to treat attacks occurring in patients with HAE-C1INH receiving LTP, including the need for a second on-demand dose

Search strategy

- **Search database:** PubMed
- **Search query:**

  ("Hereditary angioedema" OR "hereditary angioedemas" OR "hereditary angiooedema" OR "hereditary angio-edema" OR "hereditary angio-oedema" OR "hereditary angioneurotic edema" OR "hereditary angioneurotic oedema" OR "hereditary angio-neurotic edema" OR "hereditary angio-neurotic oedema")

  AND

  ("C1 esterase inhibitor" OR "C1 inhibitor" OR "C1INH" OR "C1-INH" OR "Cinryze" OR "Cetor" OR "Haegarda" OR "Lanadelumab" OR "Monoclonal antibodies" OR "Monoclonal

antibody" OR "Berotralstat" OR "Kallikrein" OR "Biologic" OR "Biologics" OR "Androgen" OR "Androgens" OR "Anabolic steroid" OR "Anabolic steroids" OR "Danazol" OR "Stanozolol" OR "Oxandrolone" OR "Methyltestosterone" OR "Aminocaproic acid" OR "6-aminohexanoic acid" OR "Amicar" OR "Tranexamic acid" OR "Treatment" OR "Treatments" OR "Therapy" OR "Therapies" OR "Therapeutic" OR "Therapeutics" OR "Prophylaxis" OR "Prophylactic" OR "Prevention" OR "Prevent" OR "Management" OR "Breakthrough")

- **PubMed search parameters:** No PubMed search parameters/filters are to be applied during the search, other than limiting the search to articles in English published in the timeframe indicated below, to allow all search results to be collected and then manually reviewed
- **Timeframe:** Past 20 years (search results published on or after January 1, 2002, to present)
- **Original search date:** May 17, 2022
- **Updated search:** To ensure that the search remains up to date, the search will be repeated in PubMed with the same search query
- **Updated search date:** May 15, 2023
    - Timeframe for the updated search is May 17,2022 to present

LTP agents to be included in the SLR

- Studies should be those that report HAE attacks in patients with HAE-C1INH (i.e., type I/II HAE) receiving LTP
- In the original SLR protocol, LTP agents were restricted to those that are FDA-approved for HAE and those that are/were used for HAE prophylaxis (as indicated in HAE clinical guidelines) but are not necessarily indicated for this in the product labels. Specifically, these treatments would be C1 esterase inhibitor protein (Cinryze® and Haegarda®), lanadelumab (Takhzyro®), berotralstat (Orladeyo®), androgen steroids (danazol, stanozolol, oxandrolone, and methyltestosterone), and antifibrinolytics (aminocaproic acid and tranexamic acid)

- In this protocol amendment (dated May 15, 2023), LTP agents that have reported phase 2/3 clinical trial findings (ie, garadacimab, donidalorsen) will also be included in the systematic search[a]
    - Investigational LTP agents that only have phase 2 findings will be discussed in a subsection of the Results on 'LTP agents in early phase clinical development'

Content sources

- Both the original search and updated search are being conducted in **PubMed** to identify articles published in peer-reviewed journals in the past 21 years (limited to articles published in English language only)
- **Congresses** will NOT be searched because abstracts and presentations are not peer reviewed, they typically do not report full data, and presentations are often not available
- **Press releases** will NOT be searched because they are not peer reviewed and only report small amounts of high-level data
- Reference lists of key articles found in the PubMed search will be checked for additional relevant articles
- Relevant articles that are not found in the PubMed search may be manually added to the final list of articles
- **Manual review:** medical writer will review each search result for relevance as follows:
    - 1) All articles identified in the search results will be reviewed at the title/abstract level
    - 2) If deemed suitable by review of the title/abstract, the full text will then be reviewed to assess for inclusion in the final set of articles
        - All excluded articles will be documented with reason for exclusion

- **The following search results will be excluded:**
    - Review, opinion, perspective, and editorial articles

        - Review articles will be flagged to allow for later retrieval. They can be checked to identify any relevant articles not found in the PubMed search
    - Guidelines (eg, clinical guidelines)
    - SLR and meta-analysis articles (to avoid capturing the same data twice)
        - SLR and meta-analysis articles on HAE attacks in patients receiving LTP will be flagged to allow for later retrieval
    - Case reports
    - Preclinical, animal, in vitro, in silico, or otherwise non-human studies
    - News and views articles, news articles, and press releases
    - Books
    - Entries where the full article is not available
    - Entries that are not available in English
    - Articles that have been retracted or are otherwise not appropriate to include
    - Duplicate articles (each article should be present only once in the list of search results)
    - Studies that do NOT report on type I/II HAE, including studies that report on HAE with normal C1 esterase inhibitor protein (formerly called type 3) and studies where it is not clear whether they are reporting on type I/II HAE
    - Studies that report patients taking only on-demand therapy or only short-term prophylaxis therapy (ie, prophylaxis taken prior to a medical, surgical, or dental procedure that may trigger an attack)
    - Studies that do not report on an efficacy outcome considered relevant for the SLR. Examples of efficacy outcomes are listed in Appendix 1

- **Updated Search**
    - In the original search [May 17, 2022], studies must have reported on attacks in patients with type I/II HAE receiving one of the following LTP agents: C1INH (Cinryze or Haegarda), lanadelumab (Takhzyro), berotralstat (Orladeyo), androgens

(danazol, stanozolol, oxandrolone, or methyltestosterone), aminocaproic acid, or tranexamic acid

  - In the updated search [May 15, 2023], studies must have reported on attacks in patients with type I/II HAE receiving one of the LTP agents listed above for the original search **OR** an investigational LTP agent (ie, currently in clinical development) which has published phase 2/3 clinical trial results available (including garadacimab and donidalorsen)[a]

Final set of included articles

- Reference lists of the final set of articles (ie, those remaining after excluding search result entries per the above defined criteria) will be checked for additional relevant articles to manually add to the final list
  - Finally, the list will be checked to ensure that each article is present only once
- These criteria should result in a list of articles reporting HAE attacks in studies of patients with type I/II HAE receiving LTP; the LTP agents should be approved or commonly used for preventing HAE attacks (based on HAE clinical guidelines) or investigational LTP agents which have published phase 2/3 efficacy results
- A PRISMA diagram will be used to report the number of PubMed search results from the original and updated searches, number of entries that were excluded/included during each search, number of entries that were manually added, and number of entries in the final list
  - Investigational LTP agents that only have phase 2 findings will be included in the PRISMA flow diagram of included studies, but as only limited data will be available, we will discuss these agents in a subsection of the Results on ‘LTP agents in early phase clinical development’

References for the Study Protocol

1. Busse PJ, Christiansen SC, Riedl MA, Banerji A, Bernstein JA, Castaldo AJ, et al. US HAEA Medical Advisory Board 2020 guidelines for the management of hereditary angioedema. J Allergy Clin Immunol Pract 2021;9(1):132-50.e3. doi:10.1016/j.jaip.2020.08.046
2. Maurer M, Magerl M, Betschel S, Aberer W, Ansotegui IJ, Aygören-Pürsün E, et al. The international WAO/EAACI guideline for the management of hereditary angioedema-the 2021 revision and update. Allergy 2022;77(7):1961-90. doi:10.1111/all.15214
3. Bork K, Anderson JT, Caballero T, Craig T, Johnston DT, Li HH, et al. Assessment and management of disease burden and quality of life in patients with hereditary angioedema: a consensus report. Allergy Asthma Clin Immunol 2021;17(1):40. doi:10.1186/s13223-021-00537-2
4. Busse P, Kaplan A. Specific targeting of plasma kallikrein for treatment of hereditary angioedema: A revolutionary decade. J Allergy Clin Immunol Pract 2022;10(3):716-22. doi:10.1016/j.jaip.2021.11.011
5. Craig TJ, Reshef A, Li HH, Jacobs JS, Bernstein JA, Farkas H, et al. Efficacy and safety of garadacimab, a factor XIIa inhibitor for hereditary angioedema prevention (VANGUARD): a global, multicentre, randomised, double-blind, placebo-controlled, phase 3 trial. Lancet 2023;401(10382):1079-90. doi:10.1016/S0140-6736(23)00350-1
6. Longhurst H. Optimum use of acute treatments for hereditary angioedema: evidence-based expert consensus. Front Med (Lausanne) 2017;4:245. doi:10.3389/fmed.2017.00245
7. Aberer W, Maurer M, Bouillet L, Zanichelli A, Caballero T, Longhurst HJ, et al. Breakthrough attacks in patients with hereditary angioedema receiving long-term prophylaxis are responsive to icatibant: findings from the Icatibant Outcome Survey. Allergy Asthma Clin Immunol 2017;13:31. doi:10.1186/s13223-017-0203-z

Appendix 1

List of example efficacy outcomes considered relevant for inclusion in the SLR:

- Proportion of patients who are attack free (primary outcome of interest)
- Rate of HAE attacks
- Proportion of patients with attacks
- Number or rate of attacks requiring on-demand therapy
- Number or rate of attacks by body location
- Percentage or responders (ie, percentage of patients who experienced a reduction in attacks beyond a specific threshold)
- Number or rate of attacks by severity
- Proportion of patients with attacks by location
- Proportion of patients with attacks by severity
- Duration of attacks
- Attack severity score/symptom severity score
- Number of days with angioedema symptoms
- Duration of attack-free period
- On-demand/rescue medication use
- Proportion of patients with attacks requiring on-demand therapy or additional medical care

Table S1. Primary studies included in the systematic review

Study identifier	First author, year	Study design	LTP agent	Dose	Comparator	No. of patients	Study population	Duration of treatment	Primary outcome	Risk of bias/quality
**RCTs and open-label extension studies**										
**pdC1INH replacement**										
NCT02052141	Aygören-Pürsün, 2019 [23]	Phase 3<br>Crossover<br>Single-blind<br>Multicenter	Cinryze (IV)	500 U<br>1000 U	NA	12	Children aged ≥6 to <12 years	2 × 12-week crossover periods	Monthly normalized number of attacks in each 12-week treatment period	High risk
NCT01005888 (LEVP2005-1 Part B)	Lumry, 2013 [21]<br>Zuraw, 2010 [42]	Phase 3<br>Crossover<br>Double-blind<br>Multicenter	Cinryze (IV)	1000 U	Placebo	24	Children aged ≥6 years and adults	2 × 12-week crossover periods	Normalized number of attacks in each 12-week treatment period	Low risk
NCT00462709 (LEVP2006-4 CHANGE-3)	Zuraw, 2012 [14]<br>Lumry, 2013 [21]<br>Zuraw, 2010 [42]<br>Baker, 2013 [22]	Phase 3<br>Open-label extension<br>Multicenter	Cinryze (IV)	1000 U	NA	146	Children aged ≥1 year and adults	Median: 248 days (up to 2.6 years)	Number of attacks during the treatment period	NA
NCT01576523	Zuraw, 2015 [15]	Phase 2<br>Open-label<br>Crossover<br>Multicenter	Berinert (SC)	1500 IU<br>3000 IU<br>6000 IU	NA	18	Adults aged ≥18 years	2 × 4-week treatment periods	Mean trough C1INH activity at week 4[a]	NA
NCT01912456 (COMPACT)	Longhurst, 2017 [9]<br>Craig, 2019 [44]<br>Li, 2019 [41]	Phase 3<br>Crossover<br>Double-blind<br>Multicenter	Berinert (SC)	40 IU/kg<br>60 IU/kg	Placebo	90	Adolescents aged ≥12 years and adults	2 × 16-week crossover periods	Time-normalized number of attacks in each 16-week treatment period	Moderate risk
NCT02316353 (COMPACT-OLE)	Craig, 2019 [43]<br>Craig, 2022 [13]<br>Bernstein, 2020 [19]<br>Levy, 2020 [18]<br>Levy, 2020 [56]	Phase 3<br>Open-label extension<br>Parallel<br>Multicenter	Berinert (SC)	40 IU/kg[b]<br>60 IU/kg[b]	NA	126	Children aged ≥6 years and adults	≥52 weeks to 140 weeks[c]	Long-term safety[d]	NA
Japanese study	Fukuda, 2023 [20]	Phase 3<br>Open-label<br>Single-arm<br>Multicenter	Berinert (SC)	60 IU/kg	Baseline (3-month period before screening) or run-in (up to 8 weeks)	9	Adolescents aged ≥12 years and adults	16 weeks	Time-normalized number of attacks/month during the treatment period; C1INH activity at week 16[e]	NA

**Lanadelumab**										
NCT02093923	Banerji, 2017 [50]	Phase 1b<br>Parallel<br>Double-blind<br>Multicenter	Lanadelumab	30 mg<br>100 mg<br>300 mg<br>400 mg	Placebo	37	Adults aged ≥18 years	6 weeks[f]	Number of attacks per week from day 8 to day 50	Moderate risk
NCT02586805 (HELP)	Banerji, 2018 [8]<br>Johnston, 2021 [57]<br>Riedl, 2020 [16]<br>Craig, 2022 [58]	Phase 3<br>Parallel<br>Double-blind<br>Multicenter	Lanadelumab	150 mg<br>300 mg (Q2W)<br>300 mg (Q4W)	Placebo	125	Adolescents aged ≥12 years and adults	26 weeks	Number of attacks in the 26-week treatment period	Low risk
NCT02741596 (HELP-OLE)	Banerji, 2022 [24]<br>Craig, 2022 [58]	Phase 3<br>Open-label extension<br>Multicenter	Lanadelumab	300 mg[g]	NA	212	Adolescents aged ≥12 years and adults	Mean (SD): 29.6 (8.2) months	Long-term safety; number of investigator-confirmed HAE attacks per month during the treatment period[h]	NA
**Berotralstat**										
NCT02870972 (ApeX-1)	Aygören-Pürsün, 2018 [33]	Phase 2<br>Parallel<br>Double-blind<br>Multicenter	Berotralstat	62.5 mg<br>125 mg<br>250 mg<br>350 mg	Placebo	77[i]	Adults aged 18 to 70 years	28 days	Number of confirmed attacks from day 8 to day 28 (the effective dosing period)	Low risk
NCT03485911 (ApeX-2)	Zuraw, 2021 [10]<br>Wedner, 2021 [35]	Phase 3<br>Parallel<br>Double-blind<br>Multicenter	Berotralstat	110 mg<br>150 mg	Placebo[j]	121[k]	Adolescents aged ≥12 years and adults[l]	48 weeks	Rate of confirmed attacks during the 24-week treatment period (part 1);<br>long-term safety (part 2)[m]	Low risk
NCT03873116 (ApeX-J)	Ohsawa, 2021 [59]	Phase 3<br>Parallel<br>Double-blind<br>Multicenter	Berotralstat	110 mg<br>150 mg	Placebo[j]	19	Adolescents aged ≥12 years and adults	52 weeks	Rate of confirmed attacks during the 24-week placebo-controlled treatment period	Low risk
NCT03472040 (ApeX-S)	Farkas, 2021 [34]	Phase 3<br>Open-label extension<br>Parallel<br>Multicenter	Berotralstat	150 mg<br>110 mg[n]	NA	227	Adolescents aged ≥12 years and adults[o]	48 weeks	Long-term safety and tolerability[p]	NA

**Investigational LTP agents**										
NCT03712228	Craig, 2022 [60]	Phase 2<br>Parallel<br>Double-blind<br>Multicenter	Garadacimab	75 mg[q]<br>200 mg[q]<br>600 mg[q]	Placebo	32	Adults aged 18 to 65 years	12-week SC administration period[r]	Time-normalized number of attacks per month during the SC treatment period	Low risk
NCT04656418 (VANGUARD)	Craig, 2023 [17]	Phase 3<br>Parallel<br>Double-blind<br>Multicenter	Garadacimab	200 mg[s]	Placebo	64	Adolescents aged ≥12 years and adults	6 months	Time-normalized number of attacks (attacks/month) during the 182-day treatment period	Low risk
NCT04030598 (ISI 721744-CS2)	Fijen, 2022 [51]	Phase 2<br>Parallel<br>Double-blind<br>Multicenter	Donidalorsen	80 mg	Placebo	20	Adults aged ≥18 years	16 weeks	Time-normalized number of attacks per month from baseline (week1) to week 17	Low risk
NCT02584959 (SAHARA)	Lumry, 2019 [61]	Phase 3,<br>Partial crossover<br>Double-blind<br>Multicenter	SHP616 (SC pdC1INH with hyaluronidase)	2000 IU	Placebo	75	Adolescents aged ≥12 years and adults[t]	2 × 14-week crossover periods or 28 weeks of active treatment	Monthly normalized number of attacks during the treatment period	Low risk
NCT02247739	Riedl, 2017 [62]	Phase 2<br>Crossover<br>Double-blind<br>Multicenter	Ruconest (recombinant C1INH)	50 IU/kg[u]	Placebo	32	Adolescents aged ≥13 years and adults	3 × 4-week crossover periods	Number of attacks in each 4-week treatment period	Low risk
Pilot study	Reshef, 2013 [63]	Phase 2<br>Open-label<br>Single-arm<br>Multicenter	Ruconest (recombinant C1INH)	50 IU/kg	NA	25	Adults aged 18 to 65 years[v]	8 weeks	Number of attacks during the treatment period	NA
**Real-world evidence and other non RCT/OLE studies**										
NCT01034969 (Icatibant Outcome Survey)	Aberer, 2017 [36]	Prospective registry	pdC1INH (IV)<br>Androgens<br>TA	NR	Patients not receiving LTP	448	Patients who have taken ≥1 dose of Firazyr[v]	Mean (SD): 3.5 (1.8) years per patient on LTP	NR	7 stars
Johannes-Gutenberg University, Germany. Semmelweis University, Hungary	Füst, 2011 [39]	Prospective cohort	Danazol	Mean:<br>136 mg (Germany)<br>128 mg (Hungary)	NR	84	Patients from Germany and Hungary[v]	≥6 years	NR	7 stars

Semmelweis University, Hungary (Grant: OTKA-NKTH 100886)	Farkas, 2013 [64]	Prospective cohort	Danazol<br>TA<br>Aminocaproic acid	Mean:<br>86 mg/day (danazol)<br>1262 mg/day (TA)	NA	48	Children aged <18 years (Hungary)	Mean:<br>6 years[w]	NA	6 stars
Angioedema Center of Reference and Excellence, Charité, Germany	Buttgereit, 2021 [65]	Prospective cohort	Lanadelumab	NR	NR	30	Adults aged 19 to 78 years[v] (Germany)	Median (range): 29.9 (3.3-65.3) weeks	NR	6 stars
Hospitals in Denmark	Rasmussen, 2016 [37]	Prospective cohort	Cinryze (IV)	1000 U	Baseline (prior to receiving LTP with C1INH)	6	Adults from Denmark[v]	Median (range): 8.5 (4-15) months	NR	6 stars
Hospitals in Switzerland	Steiner, 2016 [30]	Retrospective cohort	Danazol<br>TA	NR	NR	104	Children and adults aged 5 to 80 years[v] (Switzerland)	1 year	NR	5 stars
IAHAE-registered patients	Zanichelli, 2011 [49]	Prospective cohort	Danazol<br>Stanozolol<br>TA	Danazol: 50-200 mg<br>Stanozolol: 2-4 mg<br>TA:1.5-3 g	NR	103	Children and adults aged 5 to 93 years[v] (Italy)	NR	NR	5 stars
University Hospital of Ulm, Germany	Hahn, 2020 [46]	Prospective cohort	Lanadelumab	300 mg	Prior to enrolment (when receiving on-demand therapy only)	12	Patients aged 16 to 73 years[v] (Germany)	≥6 months	NR	4 stars
Hospitals in Australia	Katelaris, 2023 [48]	Prospective cohort	Danazol[x]<br>TA<br>C1INH (IV)[x]<br>C1INH (SC)[x]<br>Lanadelumab[x]<br>Berotralstat[y]	NR	Patients only receiving on-demand therapy	50	Adolescents aged ≥12 years and adults (Australia)	Mean: 9.1 months	NR	4 stars
University Clinics in Ulm or Munich, Germany	Greve, 2016 [66]	Prospective cohort	Cinryze	1000 U	Prior to enrollment (when receiving on-demand therapy only)	7	Adults aged 26 to 72 years[v] (Germany)	12 months	NR	3 stars

US-HAEA	Zuraw, 2016 [27]	Patient survey	Attenuated androgens	Variable	Patients who had never used androgens and patients who had previously used but discontinued androgens	650	Patients from the United States[v]	NA	NR	3 stars
United Kingdom audit	Dorr, 2023 [45]	Retrospective chart review	Lanadelumab	NR	Baseline (prior to commencing lanadelumab)	62	Patients from the United Kingdom[v]	Up to 12 months	NR	3 stars
United Kingdom audit	Ahuja, 2023 [47]	Patient survey	Berotralstat	150 mg	Baseline (3-month period prior to commencing berotralstat)	54	Patients from the United Kingdom[v]	6 months	NA	2 stars
Peking Union Medical College Hospital, China (S-K661)	Liu, 2020 [28] Xu,2022 [67]	Retrospective cohort/registry	Danazol[z] TA	Danazol: <200-600 mg[z] TA: 0.9-3 g	NA	107[z]	Children and adults aged ≥11 years[v] (China)	NR	NR	6 stars
Johannes-Gutenberg University, Germany	Bork, 2003 [30]	Retrospective chart review	Danazol	50-200 mg	NR	123	Children and adults aged ≥3 years[v] (Germany)	Mean: 7.4 years	NR	6 stars
University of Mainz, Germany University of Odense, Denmark	Bork, 2008 [38]	Retrospective chart review	Danazol	100-600 mg	Attack rate in the 3-year period prior to commencing danazol	118	Patients aged 15 to 74 years from Germany and Denmark[v]	Mean: 11.0 years	Mean number of attacks per year during the treatment period	6 stars
Hospitals in South Korea	Jung, 2018 [68]	Retrospective chart review	Danazol TA	Danazol: mean 231.5 mg TA: mean 666.7 mg	Prior to commencing LTP	65	Patients from Korea[v]	Mean (SD): 4.1 (4.6) years	NR	6 stars

Hospitals in Poland	Piotrowicz-Wójcik, 2021[69]	Patient survey	Danazol<br>TA	NR	Patients receiving on-demand treatment only	138	Adults aged ≥18 years from Poland	NR	NR	6 stars
National Reference Centre for Angioedema (CREAK)	Wintenberger, 2014 [31]	Retrospective chart review	TA	Median:<br>3 g	Baseline (6 months prior to commencing TA)	12	Patients aged ≥16 years (France)	Median:<br>34 months	NR	6 stars
Hospital Universitario La Paz, Spain	Gomez-Traseira, 2015[70]	Retrospective chart review	Danazol<br>Stanozolol<br>TA<br>Berinert (IV)	Mean weekly dose: 6.7 mg (stanozolol)<br>561.1 mg (danazol)<br>6666.7 mg (TA)<br>3250 U (Berinert)	NA	112	Children and adults aged 4 to 75 years[v] (Spain)	NR	NR	5 stars
Single-center (Location not stated)	Abuzakouk, 2022 [26]	Retrospective chart review	Lanadelumab	300 mg	Baseline (prior to commencing lanadelumab)	9	Adults aged 21 to 55 years[v] switching from another LTP agent	Median:<br>36 weeks	NR	6 stars
Academic hospitals, Canada	Iaboni, 2021[25]	Retrospective chart review	Lanadelumab	300 mg	Baseline (1 year prior to commencing lanadeluamb)	12	Adults aged 24 to 74 years[v] (Canada)	12 months	NA	5 stars
Czech national registry	Hakl, 2022 [40]	Retrospective chart review	Danazol<br>TA<br>C1INH (SC or IV pdC1INH, or IV recombinant C1INH)	NR	NA	66	Children and adults aged 5 to 74 years[v] with a laryngeal attack (Czech)	NR	NR	4 stars

HUCFF-UFRJ, Brazil	Lima, 2020 [71]	Retrospective chart review	Danazol<br>Oxandrolone<br>Aminocaproic acid<br>TA<br>C1INH (exact product and administration route not specified)	Variable	NA	10	Patients aged 16 to 70 years[v] undergoing dental surgery (Brazil)	7 days post procedure	NR	3 stars
Latvian Council of Science (lzp-2020/1-0269)	Kanepa, 2023 [72]	Physician survey	Danazol<br>TA	NR	NA	10	Adults aged 32 to 63[v] (Latvia)	NR	NR	3 stars

ATU, authorization for temporary use; *C1INH,* C1 inhibitor; *HAE,* hereditary angioedema; *HUCFF-UFRJ,* Hospital Universitario Clementino Fraga Filho of the Federal University of Rio de Janeiro; *IAHAE,* Italian Association of Patients with Hereditary Angioedema; *IV,* intravenous; *LTP,* long-term prophylaxis; *NA,* not applicable; *NR,* not reported; *Q2W,* every 2 weeks; *Q4W,* every 4 weeks; *SC,* subcutaneous; *TA,* tranexamic acid; *TEAE,* treatment-emergent adverse events; *US-HAE,* United States Hereditary Angioedema Association.

[a]The primary endpoint of mean trough C1INH functional activity were model-derived rather than observed trough levels. [b]Eligible patients were randomly assigned to receive sc-Berniert with a dose of either 40 or 60 IU/kg twice weekly for 52 weeks. However, during the first 24 weeks of treatment, patients experiencing ≥12 HAE attack per 4-week evaluation period were eligible for incremental dose increases of 20 IU/kg (up to a maximum of 80 IU/kg) at the discretion of the investigator. In the second treatment period of 28 weeks, patients experiencing ≥3 HAE attacks per 8-week evaluation period were eligible for dose increases to optimize treatment response. [c]Patients in the United States had the option to continue treatment for up to 140 weeks. [d]The primary prespecified safety endpoints were person-time incidence rates of related serious adverse events, adverse events leading to premature discontinuation, adverse events of special interest (thromboembolic events and anaphylaxis), HAE attacks resulting in hospitalization, severe injection site reactions (grade by the investigator), and the development of neutralizing anti-C1INH antibodies. [e]The study reported 2 primary endpoints. [f]Patients received 2 subcutaneous injections of study drug, given 14 days apart. The primary efficacy analysis included the number of attacks during the 6-week interval from days 8 to 50. [g]In rollover patients, a single dose of lanadelumab 300 mg was received at study entry until the patient experienced their first attack, following which the patient received lanadelumab 300 mg Q2W. Nonrollover patients received lanadelumab 300 mg Q2W from day 0. [h]The efficacy endpoint was a secondary outcome. Efficacy analyses for rollover patients were based on occurrence of attacks during the regular Q2W dosing stage and the first HAE attack during the dose-and-wait period was not counted. [i]Of the 77 patients who underwent randomization, 2 patients did not receive any study treatment. [j]In the APeX-2 and APeX-J studies, patients were randomized to berotralstat 150 mg, berotralstat 110 mg, or placebo for 24 weeks in part 1. Patients receiving placebo in part 1 were rerandomized to berotralstat 150 or 110 mg in part 2. [k]One hundred twenty-one patients were randomized and 120 were treated in part 1, and 108 patients completed dosing in part 1 and received ≥1 dose of berotralstat in part 2. [l]Patients in the United States and Canada were required to be ≥12 years of age and patients in Europe were required to be ≥18 years of age. [m]The long-term safety primary endpoints included the number and proportion of patients with TEAEs, serious TEAEs, grade 3 or 4 TEAEs or laboratory abnormalities, and rash. [n]The study was initially designed to evaluate berotralstat 150 mg over 48 weeks of treatment, but the protocol was amended to include the berotralstat 110-mg dose. [o]Patients were eligible in all participating countries if aged ≥18 years and if aged ≥12 years in certain countries. [p]Safety endpoints included the proportion of patients with TEAEs, grade 3 or 4 TEAEs or treatment-emergent laboratory abnormalities, serious TEAEs, discontinuations due to TEAEs, drug-related TEAEs consistent with drug rash. [q]Initial intravenous loading doses of placebo 40, 100, and 300 mg were administered on day 1, followed by subcutaneous treatment with placebo or 75, 200, and 600 mg of garadacimab, respectively, on day 6 and every 4 weeks thereafter for 12 weeks. [r]The primary endpoint was assessed during the 12-week SC administration period from days 6 to 63. [s]Following an initial loading dose of 400 mg of garadacimab. [t]Patients in Germany and Israel were required to be ≥18 years of age to be eligible. [u]Ruconest dosing was 50 IU/kg for patients weighing <84 kg or 4200 IU for patients weighing ≥84 kg. [v]An age range for eligibility was not stated. [w]Pediatric patients were followed up from diagnosis until 18 years of age. [x]Danazol was difficult to access during the study owing to local supply discontinuation, IV C1INH use for LTP was being used off label in Australia and was only available for patients who had ≥8 documented attacks per month, SC C1INH became available in Australia during the study and was only available for patients who had ≥8 documented attacks per month, and lanadelumab was only available through compassionate access. [y]Berotralstat was received as part of a clinical trial and was not approved for use in Australia at the time the study was conducted. [z]Liu et al reported treatment patterns in 107 patients with HAE in China and Xu et al reported on a subset of 74 patients receiving LTP with danazol.

Table S2. Attack severity in patients who received LTP in phase 3 non-placebo-controlled trials and real-world observational studies

First author, year of publication	Duration of treatment	No. of patients	Assessment of attack severity	LTP agent and dose	Attack severity, mean (SD) or n (%)		
					**Baseline**	**LTP**	***P* value**
Aygören-Pürsün, 2019 [23]	2 × 12-weeks	12	Attack severity score, mean (SD)[a]	Cinryze 500 U every 3-4 days	7.2 (6.0)	2.0 (2.9)[b]	NR
Rasmussen, 2016 [37]	4-15 months	6	Attack severity score, mean (SD)[a]	Cinryze 1000 U every 3-4 days	2.1	2.3	NR
Fukuda, 2023 [20]	16 weeks	9	Time-normalized relative reduction in moderate-to-severe attacks	Berinert 60 IU/kg twice weekly	NR	88.8% relative reduction	0.008
Dorr, 2023 [45]	Up to 12 months	62	Number of severe attacks per month, mean (SD)	Lanadelumab 300 mg Q2W	7.2 (7.2)	0.4 (1.4) 6 months	NR
						0.3 (0.7) 12 months	
Hahn, 2020 [46]	≥6 months	12	Reduction in the number of mild, moderate, and severe attacks per month	Lanadelumab 300 mg Q2W	NR[c]	Mild	0.008
						Moderate	<0.0001
						Severe	0.0001
Banerji, 2022 [24]	29.6 months (mean)	212	Number of moderate or severe attacks per 4 weeks, mean	Lanadelumab 300 mg Q2W[d]	2.0	0.2	NR
Ahuja, 2023 [47]	6 months	54	Attack severity score, mean (SD)[e]	Berotralstat 150 mg QD	3.5 (0.8)	2.3 (1.2)	<0.0001
Zuraw 2016 [28]	NA	334[f]	Attack severity score, mean (SD)[e]	Attenuated androgens	4.3 (0.1)	2.5 (0.1)	<0.0001
Aberer, 2017 [36]	3.5 years (mean)	448	Proportion of attacks rated as severe/very severe, %	C1INH	53	46	0.193
				Androgens	53	69	0.043
				TA	53	53	0.989
Katelaris, 2023 [48]	9.1 months (mean)	49	Proportion of attacks rated as severe or significant, %	SC C1INH	22	35	NR
				IV C1INH	22	23	NR
				Danazol	22	23	NR
				Tranexamic acid	22	18	NR
				Lanadelumab	22	0	NR

C1INH, C1 inhibitor; *IV*, intravenous; *LTP,* long-term prophylaxis; *NA,* not applicable; *NR,* not reported; *Q2W*, every 2 weeks; *Q4W*, every 4 weeks; *QD*, once daily; *SD*, standard deviation.

[a]Attack severity score was based on a 3-point scale, with 1 indicating mild, 2 indicating moderate, and 3 indicating severe. [b]Presented data are limited to the IV pdC1INH 500 U dose as it is the approved dose for children aged 6 to <12 years.[56] [c]The data were shown graphically by baseline; however, mean and SD values were not reported for LTP. [d]In rollover patients, a single dose of lanadelumab 300 mg was received at study entry and until the patient experienced their first attack, following which the patient received lanadelumab 300 mg Q2W. Non-rollover patients received lanadelumab 300 mg Q2W from study entry onward. [e]Attack severity score was based on a 5-point scale, with 1 indicating very mild; 2, mild; 3, moderate; 4, severe; and 5, very severe. [f]Mean (SD) baseline and on LTP treatment attack severity score among 344 patients receiving attenuated androgens at the time of the survey.

Table S3. Attack duration in phase 3 randomized placebo-controlled trials

First author, year of publication	Duration of treatment	No. of patients	LTP agent and dose	Attack duration, mean (SD)		
				LTP	Placebo	*P* value
Zuraw, 2010 [42]	2 × 12-weeks	24	Cinryze 1000 U every 3-4 days	2.1 (1.1) days	3.4 (1.4) days	0.002
Li, 2019 [41]	2 × 16-weeks	90	Berinert 40 IU/kg twice weekly	1.8 (1.1) days	2.1 (1.2) days	NR
			Berinert 60 IU/kg twice weekly	1.6 (1.0) days	1.6 (0.7) days	NR
Banerji, 2018 [8]	26 weeks	125	Lanadelumab 150 mg Q4W	35.6 (24.9) hours	33.5 (23.4) hours	0.770
			Lanadelumab 300 mg Q4W	26.0 (21.1) hours	33.5 (23.4) hours	0.222
			Lanadelumab 300 mg Q2W	26.6 (22.7) hours	33.5 (23.4) hours	0.330

LTP, long-term prophylaxis; *NR*, not reported; *Q2W*, every 2 weeks; *Q4W*, every 4 weeks; *SD*, standard deviation.

Table S4. Attack duration in real-world observational studies

First author, year of publication	Study design	No. of patients	LTP agent	Attack duration, mean		
				**Baseline**	**LTP + on-demand treatment**	***P* value**
Rasmussen, 2016 [37]	Prospective cohort	6	Cinryze	38.7 hours	20.0 hours	NR
**First author, year of publication**	**Study design**	**No. of patients**	**LTP agent**	**Attack duration, median**		
				**On-demand therapy only**	**On LTP treatment**	***P* value**
Aberer, 2017 [36]	Prospective registry (icatibant outcome survey)	448	Any LTP agent	9.0 hours	8.0 hours	0.543
			C1INH	9.0 hours	4.0 hours	0.041
			Androgens	9.0 hours	8.0 hours	0.984
			TA	9.0 hours	11.6 hours	0.016
Zanichelli, 2011 [49]	Prospective cohort	103	Attenuated androgens	1.7 days	1.5 days	NR
			Antifibrinolytics	1.7 days	1.6 days	NR

C1INH, C1 inhibitor; *LTP*, long-term prophylaxis; *NR*, not reported; *TA*, tranexamic acid.

References

1. Maurer M, Magerl M, Betschel S, Aberer W, Ansotegui IJ, Aygören-Pürsün E, et al (2022) The international WAO/EAACI guideline for the management of hereditary angioedema-the 2021 revision and update. Allergy 77:1961-1990. https://doi.org/10.1111/all.15214

2. Busse PJ, Christiansen SC, Riedl MA, Banerji A, Bernstein JA, Castaldo AJ, Craig T, Davis-Lorton M, Frank MM, Henry Li H, Lumry WR, Zuraw BL (2021) US HAEA Medical Advisory Board 2020 guidelines for the management of hereditary angioedema. J Allergy Clin Immunol Pract 9:132-50.e3. https://doi.org/10.1016/j.jaip.2020.08.046

3. Busse P, Kaplan A (2022) Specific targeting of plasma kallikrein for treatment of hereditary angioedema: a revolutionary decade. J Allergy Clin Immunol Pract 10:716-22. https://doi.org/10.1016/j.jaip.2021.11.011

4. Maurer M, Aygören-Pürsün E, Banerji A, Bernstein JA, Balle Boysen H, Busse PJ, et al (2021) Consensus on treatment goals in hereditary angioedema: a global Delphi initiative. J Allergy Clin Immunol 148:1526-1532. https://doi.org/10.1016/j.jaci.2021.05.016

5. Bork K, Anderson JT, Caballero T, Craig T, Johnston DT, Li HH, Longhurst HJ, Radojici C, Riedl M (2021) Assessment and management of disease burden and quality of life in patients with hereditary angioedema: a consensus report. Allergy Asthma Clin Immunol 17:40. https://doi.org/10.1186/s13223-021-00537-2

6. Lumry WR (2018) Hereditary angioedema: the economics of treatment of an orphan disease. Front Med (Lausanne) 16:5:22. https://doi.org/10.3389/fmed.2018.00022

7. Beard N, Frese M, Smertina E, Mere P, Katelaris C, Mills K (2022) Interventions for the long-term prevention of hereditary angioedema attacks. Cochrane Database Syst Rev 11:CD013403. https://doi.org/10.1002/14651858.CD013403.pub2

8. Banerji A, Riedl MA, Bernstein JA, Cicardi M, Longhurst HJ, Zuraw BL, et al (2018) Effect of lanadelumab compared with placebo on prevention of hereditary angioedema attacks: a randomized clinical trial. JAMA 320:2108-2121. https://doi.org/10.1001/jama.2018.16773

9. Longhurst H, Cicardi M, Craig T, Bork K, Grattan C, Baker J, et al (2017) Prevention of hereditary angioedema attacks with a subcutaneous C1 inhibitor. N Engl J Med 376:1131-1140. https://doi.org/10.1056/NEJMoa1613627

10. Zuraw B, Lumry WR, Johnston DT, Aygören-Pürsün E, Banerji A, Bernstein JA, et al (2021) Oral once-daily berotralstat for the prevention of hereditary angioedema attacks: a randomized, double-blind, placebo-controlled phase 3 trial. J Allergy Clin Immunol 148:164-172.e9. https://doi.org/10.1016/j.jaci.2020.10.015

11. Sterne JAC, Savović J, Page MJ, Elbers RG, Blencowe NS, Boutron I, et al (2019) RoB 2: a revised tool for assessing risk of bias in randomised trials. BMJ 366:l4898. https://doi.org/10.1136/bmj.l4898

12. Wells GA, Shea B, O'Connell D, Petersen J, Welch V, Losos M, Tugwell P (2021) The Newcastle-Ottawa Scale (NOS) for assessing the quality of nonrandomized studies in meta-analyses. https://www.ohri.ca/programs/clinical_epidemiology/oxford.asp. Accessed 03 December 2023.

13. Craig T, Feuersenger H, Pragst I, Dang J (2022) Prophylactic therapy with subcutaneous C1-inhibitor is associated with sustained symptom control in patients with hereditary angioedema. Allergy Asthma Proc 43:202-208. https://doi.org/10.2500/aap.2022.43.220016

14. Zuraw BL, Kalfus I (2012) Safety and efficacy of prophylactic nanofiltered C1-inhibitor in hereditary angioedema. Am J Med 125:938.e1-7. https://doi.org/10.1016/j.amjmed.2012.02.020

15. Zuraw BL, Cicardi M, Longhurst HJ, Bernstein JA, Li HH, Magerl M, Martinz-Saguer I, Rehman SMM, Staubach P, Feuersenger H, Parasrampuria R, Sidh J, Edelman J, Craig T (2015) Phase II study results of a replacement therapy for hereditary angioedema with subcutaneous C1-inhibitor concentrate. Allergy 70:1319-1328. https://doi.org/10.1111/all.12658

16. Riedl MA, Maurer M, Bernstein JA, Banerji A, Longhurst HJ, Li HH, Peng L, Hao J, Juethner S, Lumry WR, HELP Investigators (2020) Lanadelumab demonstrates rapid and sustained prevention of hereditary angioedema attacks. Allergy 75:2879-2887. https://doi.org/10.1111/all.14416

17. Craig TJ, Reshef A, Li HH, Jacobs JS, Bernstein JA, Farkas H, et al. (2023) Efficacy and safety of garadacimab, a factor XIIa inhibitor for hereditary angioedema prevention (VANGUARD):

a global, multicentre, randomised, double-blind, placebo-controlled, phase 3 trial. Lancet 401:1079-1090. https://doi.org/10.1016/S0140-6736(23)00350-1

18. Levy D, Caballero T, Hussain I, Reshef A, Anderson J, Baker J, Schwartz LB, Cicardi M, Prusty S, Feuersenger H, Pragst I, Manning ME (2020) Long-term efficacy of subcutaneous C1 inhibitor in pediatric patients with hereditary angioedema. Pediatr Allergy Immunol Pulmonol 33:136-141. https://doi.org/10.1089/ped.2020.1143

19. Bernstein JA, Schwartz L, Yang W, Baker J, Anderson J, Farkas H, Aygören-Pürsün E, Bygum A, Jacobs I, Feuersenger H, Pragst I, Riedl MA (2020) Long-term safety and efficacy of subcutaneous C1-inhibitor in older patients with hereditary angioedema. Ann Allergy Asthma Immunol 125:334-340.e1. https://doi.org/10.1016/j.anai.2020.05.015

20. Fukuda T, Yamagami K, Kawahata K, Suzuki Y, Sasaki Y, Miyagi T, Jacobs I, Lawo J, Glassman F, Akama H, Hide M, Ohsawa I (2023) Efficacy, pharmacokinetics, and safety of subcutaneous C1-esterase inhibitor as prophylaxis in Japanese patients with hereditary angioedema: results of a Phase 3 study. Allergol Int 72:451-457. https://doi.org/10.1016/j.alit.2023.02.002

21. Lumry W, Manning ME, Hurewitz DS, Davis-Lorton M, Fitts D, Kalfus IN, Uknis ME (2013) Nanofiltered C1-esterase inhibitor for the acute management and prevention of hereditary angioedema attacks due to C1-inhibitor deficiency in children. J Pediatr 162:1017-1122.e1-2. https://doi.org/10.1016/j.jpeds.2012.11.030

22. Baker JW, Craig TJ, Riedl MA, Banerji A, Fitts D, Kalfus IN, Uknis ME (2013) Nanofiltered C1 esterase inhibitor (human) for hereditary angioedema attacks in pregnant women. Allergy Asthma Proc 34:162-169. https://doi.org/10.2500/aap.2013.34.3645

23. Aygören-Pürsün E, Soteres DF, Nieto-Martinez SA, Christensen J, Jacobson KW, Moldovan D, Van Leerberghe A, Tang Y, Lu P, Vardi M, Schranz J, Martinez-Saguer I (2019) A randomized trial of human C1 inhibitor prophylaxis in children with hereditary angioedema. Pediatr Allergy Immunol 30:553-561. https://doi.org/10.1111/pai.13060

24. Banerji A, Bernstein JA, Johnston DT, Lumry WR, Magerl M, Maurer M, Martinez-Saguer I, Zanichelli A, Hao J, Inhaber N, Yu M, Riedl MA, HELP OLE Investigators (2022) Long-term

prevention of hereditary angioedema attacks with lanadelumab: the HELP OLE study. Allergy 77:979-990. https://doi.org/10.1111/all.15011

25. Iaboni A, Kanani A, Lacuesta G, Song C, Kan M, Betschel SD (2021) Impact of lanadelumab in hereditary angioedema: a case series of 12 patients in Canada. Allergy Asthma Clin Immunol 17:78. https://doi.org/10.1186/s13223-021-00579-6

26. Abuzakouk M, Ghorab O, Al-Hameli H, Salvo F, Grandon D, Maurer M (2022) Using an extended treatment regimen of lanadelumab in the prophylaxis of hereditary angioedema: a single-centre experience. World Allergy Organ J 15:100664. https://doi.org/10.1016/j.waojou.2022.100664

27. Zuraw BL, Davis DK, Castaldo AJ, Christiansen SC (2016) Tolerability and effectiveness of 17-α-alkylated androgen therapy for hereditary angioedema: a re-examination. J Allergy Clin Immunol Pract 4:948-955.e15. https://doi.org/10.1016/j.jaip.2016.03.024

28. Liu S, Xu Q, Xu Y, Wang X, Zhi Y (2020) Current status of the management of hereditary angioedema in China: a patient-based, cross-sectional survey. Eur J Dermatol 30:169-176. https://doi.org/10.1684/ejd/2020.3758

29. Bork K, Bygum A, Hardt J (2008) Benefits and risks of danazol in hereditary angioedema: a long-term survey of 118 patients. Ann Allergy Asthma Immunol 100:153-161. https://doi.org/10.1016/S1081-1206(10)60424-3

30. Steiner UC, Weber-Chrysochoou C, Helbling A, Scherer K, Grendelmeier PS, Wuillemin WA (2016) Hereditary angioedema due to C1 - inhibitor deficiency in Switzerland: clinical characteristics and therapeutic modalities within a cohort study. Orphanet J Rare Dis 11:43. https://doi.org/10.1186/s13023-016-0423-1

31. Wintenberger C, Boccon-Gibod I, Launay D, Fain O, Kanny G, Jeandel PY, Martin L, Gompel A, Bouillet L (2014) Tranexamic acid as maintenance treatment for non-histaminergic angioedema: analysis of efficacy and safety in 37 patients. Clin Exp Immunol 178:112-117. https://doi.org/10.1111/cei.12379

32. Takeda Manufacturing Austria AG. Cinryze 500 IU powder and solvent for solution for injection. Takeda Manufacturing AG: Vienna, Austria; 2022.

33. Aygören-Pürsün E, Bygum A, Grivcheva-Panovska V, Magerl M, Graff J, Steiner UC, et al (2018) Oral plasma kallikrein inhibitor for prophylaxis in hereditary angioedema. N Engl J Med 379:352-362. https://doi.org/10.1056/NEJMoa1716995

34. Farkas H, Stobiecki M, Peter J, Kinaciyan T, Maurer M, Aygören-Pürsün E, et al (2021) Long-term safety and effectiveness of berotralstat for hereditary angioedema: the open-label APeX-S study. Clin Transl Allergy 11:e12035. https://doi.org/10.1002/clt2.12035

35. Wedner HJ, Aygören-Pürsün E, Bernstein J, Craig T, Gower R, Jacobs JS, Johnston DT, Lumry WR, Zuraw BL, Best JM, Iocca HA, Murray SC, Desai B, Nagy E, Sheridan WP, Kiani-Alikhan S (2021) Randomized trial of the efficacy and safety of berotralstat (BCX7353) as an oral prophylactic therapy for hereditary angioedema: results of APeX-2 through 48 weeks (part 2). J Allergy Clin Immunol Pract 9:2305-2314.e4. https://doi.org/10.1016/j.jaip.2021.03.057

36. Aberer W, Maurer M, Bouillet L, Zanicheeli A, Caballero T, Longhurst HJ, Perrin A, Andresen I, IOS Study Group (2017) Breakthrough attacks in patients with hereditary angioedema receiving long-term prophylaxis are responsive to icatibant: findings from the Icatibant Outcome Survey. Allergy Asthma Clin Immunol 13:31. https://doi.org/10.1186/s13223-017-0203-z

37. Rasmussen ER, Aagaard L, Bygum A (2016) Real-life experience with long-term prophylactic C1 inhibitor concentrate treatment of patients with hereditary angioedema: effectiveness and cost. Ann Allergy Asthma Immunol 116:476-477. https://doi.org/10.1016/j.anai.2016.03.008

38. Bork K, Hardt J, Schicketanz KH, Ressel N (2003) Clinical studies of sudden upper airway obstruction in patients with hereditary angioedema due to C1 esterase inhibitor deficiency. Arch Intern Med 163:1229-1235. https://doi.org/10.1001/archinte.163.10.1229

39. Füst G, Farkas H, Csuka D, Varga L, Bork K (2011) Long-term efficacy of danazol treatment in hereditary angioedema. Eur J Clin Invest 41:256-262. https://doi.org/10.1111/j.1365-2362.2010.02402.x

40. Hakl R, Kuklínek P, Sobotková M, Krčmová I, Králíčová P, Vachová M, Hanzlíková J, Nováčková M, Svoboda M, Kováčová I, Litzman J (2022) Registry-based analysis of Icatibant and C1-inhibitor use in treatment of laryngeal attacks of hereditary angioedema. Clin Exp Allergy 52:994-997. https://doi.org/10.1111/cea.14182

41. Li HH, Zuraw B, Longhurst HJ, Cicardi M, Bork K, Baker J, et al (2019) Subcutaneous C1 inhibitor for prevention of attacks of hereditary angioedema: additional outcomes and subgroup analysis of a placebo-controlled randomized study. Allergy Asthma Clin Immunol 15:49. https://doi.org/10.1186/s13223-019-0362-1

42. Zuraw BL, Busse PJ, White M, Jacobs J, Lumry W, Baker J, et al (2010) Nanofiltered C1 inhibitor concentrate for treatment of hereditary angioedema. N Engl J Med 363:513-522. https://doi.org/10.1056/MEJMoa0805538

43. Craig T, Zuraw B, Longhurst H, Circardi M, Bork K, Grattan C, et al (2019) Long-term outcomes with subcutaneous C1-inhibitor replacement therapy for prevention of hereditary angioedema attacks. J Allergy Clin Immunol Pract 7:1793-1802.e2. https://doi.org/10.1016/j.jaip.2019.01.054

44. Craig T, Lumry W, Cicardi M, Zuraw B, Bernstein JA, Anderson J, et al (2019) Treatment effect of switching from intravenous to subcutaneous C1-inhibitor for prevention of hereditary angioedema attacks: COMPACT subgroup findings. J Allergy Clin Immunol Pract 7:2035-2038. https://doi.org/10.1016/j.jaip.2019.01.007

45. Dorr AD, Chopra C, Coulter TI, Dempster J, Dziadzio M, El-Shanawany T, et al (2023) Lanadelumab for the prevention of hereditary angioedema attacks: a real-world UK audit. Allergy 78:1369-1371. https://doi.org/10.1111/all.15620

46. Hahn J, Trainotti S, Wigand MC, Schuler PJ, Hoffmann TK, Greve J (2020) Prospective analysis in patients with HAE under prophylaxis with lanadelumab: a real-life experience. J Drugs Dermatol 19:978-983. https://doi.org/10.36849/JDD.2020.5269

47. Ahuja M, Dorr A, Bode E, Boulton APR, Buckland M, Chee S, et al (2023) Berotralstat for the prophylaxis of hereditary angioedema-real-world evidence data from the United Kingdom. Allergy 78:1380-1383. https://doi.org/10.1111/all.15641

48. Katelaris CH, Boicos K, Button PH, McCloud PI, Burton PK, Perram FA, Youssef S, Tognarini D (2023) Living with hereditary angioedema in Australia: findings from a national observational study using short message service to monitor the burden of disease. J Allergy Clin Immunol Pract 11:2457-67.e1. https://doi.org/10.1016/j.jaip.2023.02.037

49. Zanichelli A, Vacchini R, Badini M, Penna V, Cicardi M (2011) Standard care impact on angioedema because of hereditary C1 inhibitor deficiency: a 21-month prospective study in a cohort of 103 patients. Allergy 66:192-196. https://doi.org/10.1111/j.1398-9995.2010.02433.x

50. Banerji A, Busse P, Shennak M, Lumry W, Davis-Lorton M, Wedner HJ, et al (2017) Inhibiting plasma kallikrein for hereditary angioedema prophylaxis. N Engl J Med 376:717-728. https://doi.org/10.1056/NEJMoa1605767

51. Fijen LM, Riedl MA, Bordone L, Bernstein JA, Raasch J, Tachdjian R, Craig T, Lumry WR, Manning ME, Alexander VJ, Newman KB, Revenko A, Baker BF, Nanavati C, Macleod AR, Schneider E, Cohn DM (2022) Inhibition of prekallikrein for hereditary angioedema. N Engl J Med 386:1026-1133. https://doi.org/10.1056/NEJMoa2109329

52. Minafra FG, Cunha LAO, Mariano RGS, Goebel GA, de Lima LS, Pinto JA (2022) Investigation of mortality of hereditary angioedema in a reference center in Brazil. J Allergy Clin Immunol Pract 10:1805-1812. https://doi.org/10.1016/j.jaip.2022.04.030

53. Riedl MA, Banerji A, Manning ME, Burrell E, Joshi N, Patel D, Machnig T, Tai M, Watson DJ (2018) Treatment patterns and healthcare resource utilization among patients with hereditary angioedma in the United States. Orphanet J Rare Dis 13:180. https://doi.org/10.1186/s13023-018-0922-3

54. Petersen RS, Fijen LM, Cohn DM (2024) Efficacy outcomes in trials with prophylactic hereditary angioedema therapy: A systematic review. Allergy 79:1345-1348. https://doi.org/10.1111/all.15962

55. Peterson RS, Fijen LM, Apfelbacher C, Magerl M, Weller K, Aberer W (2024) A core outcome set for efficacy of acute treatment of hereditary angioedema. J Allergy Clin Immunol Pract 12:1614-1621. https://doi.org/10.1016/j.jaip.2024.04.007

56. Levy DS, Farkas H, Riedl MA, Hsu FI, Brooks JP, Cicardi M, Feuersenger H, Pragst I, Reshef A (2020) Long-term efficacy and safety of subcutaneous C1-inhibitor in women with hereditary angioedema: subgroup analysis from an open-label extension of a phase 3 trial. Allergy Asthma Clin Immunol 16:8. https://doi.org/10.1186/s13223-020-0409-3

57. Johnston DT, Busse PJ, Riedl MA, Maurer M, Anderson J, Nurse C, Inhaber N, Yu M, Banerji A, HELP Study Investigators (2021) Effectiveness of lanadelumab for preventing hereditary angioedema attacks: subgroup analyses from the HELP study. Clin Exp Allergy 51:1391-1395. https://doi.org/10.1111/cea.13974

58. Craig TJ, Zaragoza-Urdaz RH, Li HH, Yu M, Ren H, Juethner S, Anderson J, HELP and HELP OLE Study Investigators (2022) Effectiveness and safety of lanadelumab in ethnic and racial minority subgroups of patients with hereditary angioedema: results from phase 3 studies. Allergy Asthma Clin Immunol 18:85. https://doi.org/10.1186/s13223-022-00721-y

59. Ohsawa I, Honda D, Suzuki Y, Fukuda T, Kohga K, Morita E, et al (2021) Oral berotralstat for the prophylaxis of hereditary angioedema attacks in patients in Japan: a phase 3 randomized trial. Allergy 76:1789-1799. https://doi.org/10.1111/all.14670

60. Craig TJ, Zaragoza-Urdaz RH, Li HH, Yu M, Ren H, Juethner S, Anderson J, HELP and HELP OLE Study Investigators (2022) Effectiveness and safety of lanadelumab in ethnic and racial minority subgroups of patients with hereditary angioedema: results from phase 3 studies. Allergy Asthma Clin Immunol 18:85. https://doi.org/10.1186/s13223-022-00721-y

61. Lumry WR, Martinez-Saguer I, Yang WH, Bernstein JA, Jacobs J, Moldovan D, Riedl MA, Johnston DT, Henry Li H, Tang Y, Schranz J, Lu P, Vardi M, Farkas H, SAHARA Study Group (2019) Fixed-dose subcutaneous C1-inhibitor liquid for prophylactic treatment of C1-INH-HAE: SAHARA randomized study. J Allergy Clin Immunol Pract 7:1610-1618.e4. https://doi.org/10.1016/j.jaip.2019.01.021

62. Riedl MA, Grivcheva-Panovska V, Moldovan D, Baker J, Yang WH, Giannetti BM, Reshef A, Andrejevic S, Lockey RF, Hakl R, Kivity S, Harper JR, Relan A, Cicardi M (2017) Recombinant human C1 esterase inhibitor for prophylaxis of hereditary angio-oedema: a phase 2, multicentre, randomised, double-blind, placebo-controlled crossover trial. Lancet 390:1595-1602. https://doi.org/10.1016/S0140-6736(17)31963-3

63. Reshef A, Moldovan D, Obtulowicz K, Leibovich I, Mihaly E, Visscher S, Relan A (2013) Recombinant human C1 inhibitor for the prophylaxis of hereditary angioedema attacks: a pilot study. Allergy 68:118-124. https://doi.org/10.1111/all.12060

64. Farkas H, Csuka D, Zotter Z, Varga L, Füst G (2013) Prophylactic therapy in children with hereditary angioedema. J Allergy Clin Immunol 131:579-582.e1-2. https://doi.org/10.1016/j.jaci.2012.08.001

65. Buttgereit T, Vera C, Weller K, Gutsche A, Grekowitz EM, Aykanat S, Wahn V, Krüger R, Maurer M, Magerl M (2021) Lanadelumab efficacy, safety, and injection interval extension in HAE: a real-life study. J Allergy Clin Immunol Pract 9:3744-3751. https://doi.org/10.1016/j.jaip.2021.04.072

66. Greve J, Hahn J, Nordmann M, Schuler PJ, Bas M, Hoffmann TK, Hajdu Z, Buchberger M, Strassen U (2016) Nanofiltrated C1-esterase-inhibitor in the prophylactic treatment of bradykinin-mediated angioedema. Transfusion 56:1022-1029. https://doi.org/10.1111/trf.13462

67. Xu Y, Zhi Y (2022) Long-term prophylaxis of hereditary angioedema with danazol. Chin Med J (Engl) 135:2642-2643. https://doi.org/10.1097/CM9.0000000000002144

68. Jung JW, Suh DI, Park HJ, et al (2018) Clinical features of hereditary angioedema in Korean patients: a nationwide multicenter study. Int Arch Allergy Immunol 176:272-279. https://doi.org/10.1159/000488350

69. Piotrowicz-Wójcik K, Bulanda M, Juchacz A, Jamróz-Brzeska J, Gocki J, Kuziemski K, Pawlowicz R, Porebski G (2021) Clinical characteristics and management of angioedema attacks in polish adult patients with hereditary angioedema due to C1-inhibitor deficiency. J Clin Med 10:5609. https://doi.org/10.3390/jcm10235609

70. Gómez-Traseira C, Pérez-Fernández E, López-Serrano MC, García-Ara MC, Pedrosa M, López-Trascasa M, Caballero T (2015) Clinical pattern and acute and long-term management of hereditary angioedema due to C1-esterase inhibitor deficiency. J Investig Allergol Clin Immunol 25:358-364.

71. Lima BC, Ragon CST, Veras RA, Gomes AOF, Alonso MLO, Valle SOR, Torres SR, Agostini M (2020) Hereditary angioedema: report of the dental treatment of 12 Brazilian patients. Oral Surg Oral Med Oral Pathol Oral Radiol 130:651-658. https://doi.org/10.1016/j.oooo.2020.08.038

72. Kanepa A, Nartisa I, Rots D, Gailite L, Farkas H, Kurjane N (2023) National survey on clinical and genetic characteristics of patients with hereditary angioedema in Latvia. Allergy Asthma Clin Immunol 19:28. https://doi.org/10.1186/s13223-023-00783-6
